# Temporal migration rates affect the genetic structure of populations in the biennial *Erysimum mediohispanicum* with reproductive asynchrony

**DOI:** 10.1093/aobpla/plaa037

**Published:** 2020-07-25

**Authors:** A Jesús Muñoz-Pajares, Mohamed Abdelaziz, F Xavier Picó

**Affiliations:** 1 Departamento de Genética, Universidad de Granada, Granada, Spain; 2 Research Center in Biodiversity and Genetic Resources (CIBIO), Campus Agrário de Vairão, Vairão, Portugal; 3 Biological and Environmental Sciences, School of Natural Sciences, University of Stirling, Stirling, UK; 4 Departamento de Ecología Integrativa, Estación Biológica de Doñana (EBD), Consejo Superior de Investigaciones Científicas (CSIC), Sevilla, Spain

**Keywords:** Biennials, *Erysimum mediohispanicum*, genetic diversity, genetic structure, nuclear microsatellites, simulation model, temporal migration rate

## Abstract

Migration is a process with important implications for the genetic structure of populations. However, there is an aspect of migration seldom investigated in plants: migration between temporally isolated groups of individuals within the same geographic population. The genetic implications of temporal migration can be particularly relevant for semelparous organisms, which are those that reproduce only once in a lifetime after a certain period of growth. In this case, reproductive asynchrony in individuals of the same population generates demes of individuals differing in their developmental stage (non-reproductive and reproductive). These demes are connected by temporal migrants, that is, individuals that become annually asynchronous with respect to the rest of individuals of their same deme. Here, we investigated the extent of temporal migration and its effects on temporal genetic structure in the biennial plant *Erysimum mediohispanicum*. To this end, we conducted two independent complementary approaches. First, we empirically estimated temporal migration rates and temporal genetic structure in four populations of *E. mediohispanicum* during three consecutive years using nuclear microsatellites markers. Second, we developed a demographic genetic simulation model to assess genetic structure for different migration scenarios differing in temporal migration rates and their occurrence probabilities. We hypothesized that genetic structure decreased with increasing temporal migration rates due to the homogenizing effect of migration. Empirical and modelling results were consistent and indicated a U-shape relationship between genetic structure and temporal migration rates. Overall, they indicated the existence of temporal genetic structure and that such genetic structure indeed decreased with increasing temporal migration rates. However, genetic structure increased again at high temporal migration rates. The results shed light into the effects of reproductive asynchrony on important population genetic parameters. Our study contributes to unravel the complexity of some processes that may account for genetic diversity and genetic structure of natural populations.

## Teaser

Many plants require two or more years to produce flowers, but flowering individuals can be observed every year in natural populations. This is due to ‘temporal migrants’, that is, individuals flowering apart (at a different time) from the majority of their contemporaries. In this work, we explore the consequences of temporal migration on the genetic diversity of these populations. For the first time, we have reported a U-shape relationship, meaning that intermediate migration rates maximize genetic differences between flowering and non-flowering individuals. Our study contributes to unravel the complexity of some processes shaping genetic diversity of natural populations.

## Introduction

Population is a concept with several definitions that still generates vivid discussions in biological sciences (Jonckers 1973; Waples and Gaggiotti 2006; Hey 2011). Intuitively, we can describe a population as a group of co-occurring, interbreeding individuals that pass their genetic features on to the next generation. A common characteristic of any population is that it inexorably exhibits variation in the number of individuals contributing genetically to the next generation (i.e. the effective population size). This attribute determines spatio-temporal variation in allele frequency and shapes genetic structure (Wright 1951, 1965; Kimura and Weiss 1964; Rosenberg *et al.* 2002). The most common usage of genetic structure data deals with the demographic and evolutionary history of populations across wide geographic areas, which allows the detection of major large-scale genetic patterns. In other words, the role that population dynamics, migration, adaptive variation and genetic drift plays in determining the extent of genetic structure across space (Loveless and Hamrick 1984; Slatkin 1985, 1987; Rosenberg *et al.* 2002; Manel *et al.* 2003; Storfer *et al.* 2007; Dionne *et al.* 2008).

The mentioned evolutionary and demographic processes structure populations spatially but also temporally. Recent studies on annuals comparing population genetic attributes in different points in time provided insight into how fast and how much plant populations can modify their genetic composition and genetic structure over several generations (Franks *et al.* 2007, 2016; Franks and Weis 2008; Nevo *et al.* 2012; Sultan *et al.* 2013; Bustos-Segura *et al.* 2014; Thomann *et al.* 2015; Welt *et al.* 2015; Horgan-Kobelski *et al.* 2016; Kuester *et al.* 2016; Frachon *et al.* 2017; Gómez *et al.* 2018). Such a temporal dimension in the process of genetic differentiation in plant populations can even acquire a higher degree of complexity when considering variation in key life-history traits, such as reproduction at the within-population scale (Wells and Wells 1980; Loveless and Hamrick 1984). For example, a decline in the mate probability among individuals of the same population varying in reproductive time may lead to isolation by time. In this case, individuals showing early and late reproductive times would be the extremes of a reproductive time gradient, which should exhibit the largest genetic differences (Hendry and Day 2005). Individuals with intermediate reproductive time values within this gradient would increase gene flow between the above-mentioned extremes. However, a gradient in reproduction times is not the only mechanism to generate assortative mating. This is because seed banks may also generate similar consequences on genetic structure by incorporating to the mating arena individuals from previous generations with different reproductive times (Nunney 2002; Vitalis *et al.* 2004; Dolan *et al.* 2008; Königer *et al.* 2012; Falahati-Anbaran *et al.* 2011, 2014).

**Figure 1.**
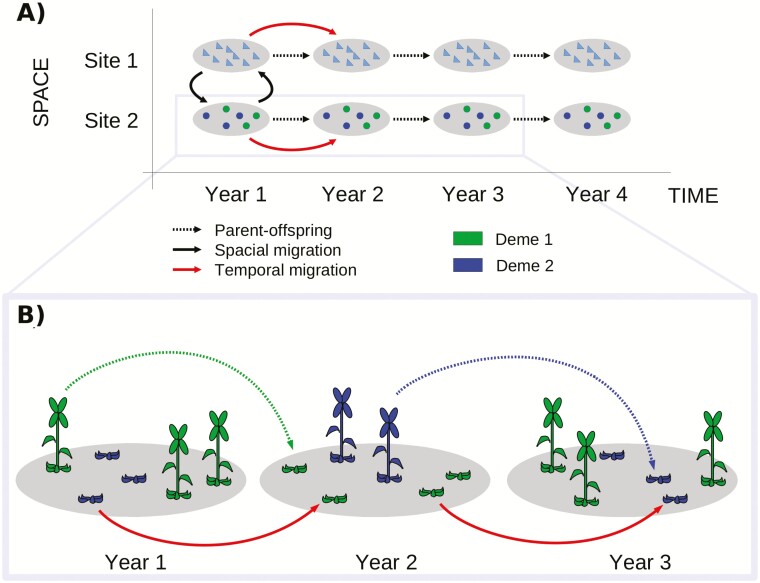
Graphical representation of the conceptual study system: a biennial organism’s life cycle. (A) Depiction of two geographically separated populations over 4 years. The genetic properties of every site and year depend on the previous generation through parent–offspring inheritance (dashed lines), the spatial migration from one geographic site to another (solid black line) and the temporal migration from 1 year to another within the same geographic site (solid red line). (B) Details on temporal migration. Individuals in a single population flowering in odd and even years (green and blue, respectively) are defined as different demes. Reproductive plants of deme #1 in year #1 produce rosettes in year #2 (dashed line). Vegetative rosettes in year #1 have two options: they can become reproductive in year #2 (thus contributing to deme #2) or remain as vegetative until year #3 (thus representing a temporal migrant and contributing to deme #1, solid red line).

Temporal variation in reproductive time will surely be more important in organisms requiring >1 year between reproductive periods. Among them, biennial species are the simplest example. Such organisms are able to show asynchrony at larger time scales (e.g. between years) in addition to the within-year asynchrony described above for isolation by time. Consequences of this reproductive asynchrony will depend on how heritable reproduction times are (Hendry *et al.* 1999; Fox 2003; Weis and Kossler 2004). Thus, in the case of complete heritability, individuals inhabiting a given geographic location are subdivided into groups of mating (demes hereafter) that would never inbreed among them, favouring the assortative mating and thus stratifying populations (Fox 2003; Weis and Kossler 2004). In the case in which reproduction time shows a significant environmental component, some individuals from a given deme may behave, for example, as triennial instead of as biennial, contributing to gene flow between demes. In that sense, individuals belonging to one deme but reproducing with another would act as temporal migrants reducing deme differentiation (Fig. 1).

In this context of temporal genetic structure, individuals from different demes of the same population co-occur in the same physical site, and developmental variation in reproduction—and probably microhabitat conditions (Denney *et al.* 2020)—determines the amount of temporal migrants. Just as with geographic migration, demes within a population may structure over time if migration rates among demes remain low. In the case of semelparous organisms, those that reproduce only once in a lifetime after a certain period of growth, the effects of temporal variation in reproduction between demes can be especially dramatic because individuals will have no more chances to pass on their genes after their unique reproductive event. According to their life histories, we could expect reproductive individuals of biennial semelparous organisms to be detected either in odd or even years. However, reproductive individuals commonly occur every year in populations of most biennial semelparous species (Kelly 1985; De Jong and Klinkhamer 1988; Petrů 2005; Valverde *et al.* 2016). The evolutionary consequences of temporal asynchrony on population differentiation has widely been studied in animals, particularly in salmonids (Aspinwall 1974; Waples 1990a, b, 2002, 2006), but examples in plant species are rather scarce (Wells and Wells 1980; Valverde *et al.* 2016). So far, most of the attention paid to temporal asynchrony in plants focused on early and late flowering time, the role of seed banks and their effects on assortative mating, population structure and speciation (Hossaert-McKey *et al.* 1996; Heath *et al.* 2002; Chung *et al.* 2003; Fox 2003; Weis and Kossler 2004; Hendry and Day 2005; Kent *et al.* 2007; Martínez-Cruz *et al.* 2007; Lundemo *et al.* 2009; Riccioni *et al.* 2010; Falahati-Anbaran *et al.* 2011, 2014; Gomaa *et al.* 2011; Sloop *et al.* 2011; Königer *et al.* 2012).

Here, we quantified the consequences of temporal migration for genetic structure of plant populations over time. To this end, we conducted two independent complementary approaches using a monocarpic plant with a biennial life history. First, we collected data from natural populations of the biennial plant *Erysimum mediohispanicum* to estimate temporal migration rates and temporal genetic structure. We estimated temporal migration rates by monitoring the fate of 1-year-old vegetative rosettes from *E. mediohispanicum* populations over several years. To assess temporal genetic structure, we genotyped with polymorphic nuclear microsatellites (Muñoz-Pajares *et al.* 2011) vegetative and reproductive *E. mediohispanicum* individuals, representing the two different demes co-occurring at each population over years. Second, we developed a simulation model to explore the population dynamics and population genetics of a biennial plant and analyse the effects of varying temporal migration rates on population genetic differentiation between two demes. In particular, the aim of this work is to address the following questions: (i) Do demes within a geographic population show significant genetic differentiation? (ii) Does temporal migration rate reduce between-demes differentiation? Overall, the results highlight the intricacy of ecological and biological factors accounting for the genetic structure of plant populations.

## Methods

### Empirical demographic and genetic data

**Figure 2.**
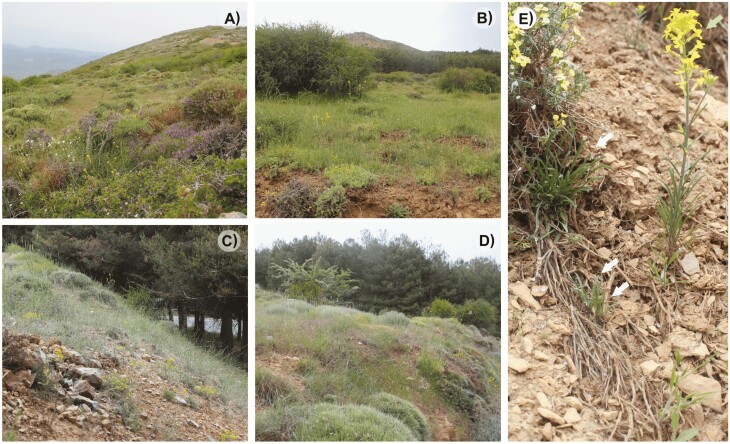
Panoramic view of the populations included in the present study. (A) Em25, 2064 m a.s.l.; (B) Em23, 1874 m a.s.l.; (C) Em01, 1750 m a.s.l.; (D) Em08, 1690 m a.s.l. Flowering plants of *E. mediohispanicum* can be observed in every picture. (E) *Erysimum mediohispanicum* flowering plants (even year, green arrow) co-occurring with vegetative plants (odd year, white arrows). Note the different rosette sizes showed by the vegetative plants in the picture.

In this study, we obtained empirical data from *E. mediohispanicum* (Brassicaceae), which is an endemic but common herb of the Iberian Peninsula exhibiting a biennial habit (Fig. 2). The plant can usually grow for 2–3 years as vegetative rosettes before dying after reproduction (Gómez 2005). Hence, *E. mediohispanicum* exhibits variation in age at first reproduction, which is the trait required to generate temporal migrants among demes as defined above. *Erysimum mediohispanicum* has a mixed mating system and a wide array of insects differing in morphology, size and behaviour (Gómez *et al.* 2007) pollinating the plant. Wind is the only known seed disperser and no seed bank has been reported for this species (Gómez 2007).

We collected data from four large *E. mediohispanicum* sites at Sierra Nevada National Park (SE Spain), separated by 0.33–1.45 km and coded as Em01, Em08, Em23 and Em25 (see Gómez *et al.* 2007 for further details of study sites). In June–July 2010, 2011 and 2012, we collected leaf tissue from 30–40 vegetative rosettes (entire above-ground rosettes) and 30–34 reproductive individuals (rosette and caulinar leaves) at each sampling site. In Em25 in 2012, we did not find vegetative rosettes and only reproductive individuals represented this sampling site for this particular year. Every year, samples were immediately stored in cellophane bags, labelled and dried with Silica Gel until DNA extraction in the laboratory.

During field campaigns, we haphazardly tagged with numbered metal plates 100–120 vegetative rosettes at each sampling site per year to estimate the proportion of rosettes that completed the life cycle in 2 years. We estimated the proportion of rosettes that required >2 years to become reproductive individuals, representing an estimation of the temporal migration rate between demes. All tagged vegetative rosettes were of similar size (~40–50 mm diameter and 10–15 leaves). We purposely discarded too small or too large rosettes to avoid merging individuals from different demes. Based on previous field observations on *E. mediohispanicum*, we considered that all tagged rosettes of similar size probably belonged to the same deme germinated in summer/autumn the year before. In other words, all tagged vegetative rosettes were 1-year-old individuals. We surveyed all tagged rosettes 1 year later to record their status including survival and their developmental stage (vegetative or reproductive). Overall, we tagged and monitored 807 *E. mediohispanicum* vegetative rosettes.

In addition, we collected leaf material from 30 individuals per deme to quantify genetic diversity and differentiation. Because our sampling design includes 3 years, four populations and two demes per population, we collected tissue from 720 individuals. We extracted DNA from all samples using DNeasy Plant Mini Kit (Qiagen, Hilden, Germany) and stored them at −80 °C until genotyping with nine nuclear microsatellite markers (codes: C5, D2, D4, D10, D11, E4, E5, E6 and E8). These markers previously showed polymorphism in *E. mediohispanicum* and other genetic properties appropriate for population genetic studies (Muñoz-Pajares *et al.* 2011). Polymerase chain reactions (PCRs) were performed in 25 μL of reaction mixture containing 2.5 μL of Buffer (10×), 1 μL of MgCl_2_ (50 mM), 0.2 μL of dNTPs (25 mM), 1 μL of forward and reverse primers (50 ng μL^−1^), 5 μL of DNA (2 ng μL^−1^), 0.15 μL of *Taq* polymerase (5U μL^−1^) and 15.15 μL of Milli-Q water. Forward primers were fluorescently tagged with 6-FAM™ (C5, E5 and E8), NED™ (D4, D10 and E6) and VIC® (D2, D11 and E4) labels (Applied Biosystems, Foster City, CA, USA). Polymerase chain reactions were performed in a Biometra Gradient Thermal Cycler (Biometra, Göttingen, Germany). Thermocycler conditions consisted of an initial 2-min denaturation at 94 °C followed by 35 cycles of denaturing for 1 min at 94 °C, annealing for 1 min at primer-specific annealing temperatures, extension for 2 min at 72 °C and a final extension step of 10 min at 72 °C. Annealing temperatures were 54 °C for D2 and E5, and 56 °C for the rest of microsatellites. The fluorescently tagged PCR products were sized using GeneScan™ LIZ 500 size standard (Applied Biosystems, Foster City, CA, USA) on an ABI 3130 Genetic Analyser (Applied Biosystems, Foster City, CA, USA). Alleles were called by the same person with Peak Scanner v.1.0 (Applied Biosystems, Foster City, CA, USA).

We found some *E. mediohispanicum* individuals with more than two alleles at few particular markers, probably because of partial genome duplication. This finding is in accordance with the genome size variation previously described for this species (Muñoz-Pajares *et al.* 2018). Specifically, the extent of duplication was rather low in this set of individuals as only 3–19 individuals per marker (0.4–2.7 %) had three or four alleles per microsatellite. In these particular cases, we selected the two most common alleles within the population among the different alleles observed per marker and individual. By doing so, we kept missing values low and avoided overestimation of low frequency alleles in our data set.

We successfully genotyped 228, 239 and 220 *E. mediohispanicum* individuals in 2010, 2011 and 2012, respectively, totalling 687 individuals. All individuals included in this study amplified successfully in at least six of nine microsatellites. Overall, our data set included 3.4 % of missing values. We genotyped twice 33 and 21 individuals from 2010 and 2011, respectively, with all microsatellites to estimate the genotyping error per locus that was of 0.1 %. Repeated genotyping indicated that genotyping error was due to allelic dropout in heterozygous loci, as usual in microsatellite genotyping (Hoffman and Amos 2005; Pompanon *et al.* 2005).

Genetic data for each site, year and developmental stage (group of reproductive and vegetative individuals) were analysed with FSTAT v.2.9.3 (Goudet 1995) and GenAlEx v.6 (Peakall and Smouse 2006) to calculate the mean number of alleles per locus (*n*_a_), mean observed heterozygosity (*H*_O_) and mean gene diversity (*H*_S_). To estimate the proportion of the total genetic variation explained by each of these levels we performed a hierarchical analysis of molecular variance as implemented in the ‘hierfstat’ package in R (Goudet 2013). The significance level of the hierarchical *F*-statistics was calculated from 1000 bootstrap permutations.

To evaluate the temporal consistency of the genetic structure found between demes within a population, we performed hierarchical analyses of molecular variance separately for different years. We estimated genetic differentiation between reproductive and vegetative groups of individuals for each site independently for 2010, 2011 and 2012 using Arlequin v.3.5.1.2 (Excoffier and Lischer 2010). All *F*_ST_ statistics (Weir and Cockerham 1984) and their significance levels were calculated from 1000 permutations.

The genetic structure among all 687 *E. mediohispanicum* individuals was estimated using the Bayesian clustering method implemented in STRUCTURE v.2.3.4 (Pritchard *et al.* 2000; Falush *et al.* 2003). All individuals were genetically different from each other, so they were non-redundant multilocus genotypes. Each run consisted of 50 000 burn-in Markov Chain Monte Carlo (MCMC) iterations and 150 000 MCMC after-burning repetitions for parameter estimation. To determine the *K* number of ancestral populations and the ancestry membership proportions of each individual, we run the algorithm 15 times for each *K* value from 1 to 15 and apply the Evanno method (Evanno *et al.* 2005) as implemented in the Structure Harvester Server (http://taylor0.biology.ucla.edu/structureHarvester/). We also applied the same procedure for every population separately, ranging *K* value from 1 to 6 in this case.

Genetic distance between demes was estimated based on the Discriminant Analysis of Principal Components (DAPC; Jombart *et al.* 2010), available in the R package adegenet v.2.1.1 (Jombart 2008), after retaining the first 50 principal component dimensions and the first two discriminant dimensions. Euclidean distances between centroids of demes per population and year indicated the genetic distance between them and therefore represented another measure of genetic structure due to temporal migration.

### The demographic genetic model

A time lag of 2 years between seed germination and sexual reproduction characterizes the lifespan of biennial plants. In populations of a biennial plant, both vegetative and reproductive individuals can occur simultaneously. Obviously, vegetative and reproductive individuals cannot interbreed because of their different developmental stages. Reproductive individuals come from seeds germinated 2 years back, whereas vegetative individuals come from seeds germinated just 1 year back. Hence, these populations encompass two distinct demes of reproductive individuals separated temporally by a single year (Fig. 1). Some individuals can show departures from this pattern, such as variation in age at first reproduction, representing migration events between demes. Ecological and genetic factors underlying reproductive asynchrony determine the occurrence of temporal migration events. Depending on these factors, migration events in natural populations may occur every year or be an extremely rare event. To explore if the evolutionary consequences of both scenarios are equivalent, we split temporal migration into two components. First, we defined *temporal migration rate* as the proportion of individuals from a given deme and year reproducing in the other deme 1 year later. Second, we define *temporal migration event probability* as the frequency at which a migration event occurs over time. Based on this, strict biennials have no temporal migration rates or no temporal migration event probabilities, whereas facultative biennials typically exhibit temporal migration rates and temporal migration event probabilities higher than zero.

We developed a basic demographic model to compute total deme size over time (*N*_T_) taking into account the total number of reproductive individuals recruited into a deme, via sexual reproduction (*N*_R_) or immigration (*N*_M_), and the number of individuals emigrating (*N*_E_) from that deme. This basic demographic model has the following structure:


$$\begin{array}{l} N_{T}=N_{R}+N_{M}-N_{E} \end{array}$$ (1)

This equation was applied to each of the two demes *d*1 and *d*2. For each deme, reproductive events occurred every 2 years in a way that when one deme is in a vegetative stage the other is reproducing, and vice versa. Hence, the *t*th generation occurred at time *y* for deme *d*1 and at time *z* for deme *d*2, which was described as:


$$\begin{array}{l} y=2t-1 \end{array}$$ (2)

$$\begin{array}{l} z=2t \end{array}$$ (3)

For each of the two demes *d*1 and *d*2, the number of individuals recruited by sexual reproduction, *N*_R_, in years *y* and *z*, respectively, was computed as follows:


$$\begin{array}{l} N_{R}(y,d1)=\lambda (y,d1)\times N_{T}(y-2,d1) \end{array}$$ (4)

$$\begin{array}{l} N_{R}(z,d2)=\lambda (z,d2)\times N_{T}(z-2,d2) \end{array}$$ (5)

where *λ*(*y*, *d*1) and *λ*(*z*, *d*2) are deme growth rates for deme *d*1 in year *y* and deme *d*2 in year *z*, respectively. Such *λ* values simulated random fluctuations of deme size sampling from a normal distribution with mean 1 and standard deviation 0.01. Although such distribution parameter values are arbitrary, they generated a stable population with slight fluctuations over time. We performed previous simulations with the same mean and different standard deviations to confirm the expected effect of increasing variation on increasing extinction rates (results not shown). Our model only explored the genetic effects of temporal isolation and migration between demes within a demographically stable population.

For each of the two demes *d*1 and *d*2, the number of emigrating individuals, *N*_E_, in years *y* and *z*, respectively, was computed as follows:


$$\begin{array}{l} N_{E}(y,d1)=m_{12}(y)\times N_{R}(y,d1) \end{array}$$ (6)

$$\begin{array}{l} N_{E}(z,d2)=m_{21}(z)\times N_{R}(z,d2) \end{array}$$ (7)

where *m*_12_(*y*) and *m*_21_(*z*) is the migration rate of vegetative individuals from deme *d*1 to deme *d*2 in year *y*, and from deme *d*2 to deme *d*1 in year *z*, respectively.

Finally, for each of the two demes *d*1 and *d*2, the number of recruited individuals via immigration, *N*_M_, in years *y* and *z*, respectively, was computed as follows:


$$\begin{array}{l} N_{M}(y,d1)=m_{21}(z-2)\times N_{R}(z-2,d2) \end{array}$$ (8)

$$\begin{array}{l} N_{M}(z,d2)=m_{12}(y)\times N_{R}(y,d1) \end{array}$$ (9)

Note that because we only have two demes, emigrating individuals from one deme in a given year corresponded to immigrating individuals to the other deme right in the next year. Given the definition of reproductive year for deme *d*1 and *d*2 (Equations 2 and 3), immigrants of *d*2 in year *z* were emigrants from *d*1 in year *y* (Equation 9). In contrast, immigrants of *d*1 in year *y* were emigrants from *d*2 in year *z* − 2 (Equation 8) **[see****Supporting Information—Table S1****]**.

For each deme, the model ran for 100 generations. A detailed exploration of model performance at each generation indicated that initial conditions did not affect the model, and that 100 generations were enough to accurately estimate all genetic parameters of interest **[see****Supporting Information—Fig. S1****]**. Model simulations encompassed 10 different scenarios with constant migration rates ranging from 0 to 90 % of individuals migrating between demes. For each migration rate, the model generated 11 additional scenarios simulating different migration event probabilities (from 0 to 1) over the 100 generations. The no-migration scenario represented the baseline simulation and showed the demographic behaviour of the simulated population. This simulation schedule was repeated 100 times totalling 11 000 runs (10 migration rates × 11 migration event probabilities × 100 replicates). The model started with 1000 initial individuals from each deme. Simulated individuals can only interbreed either when they are 2 years old or when they are 3 years old if they have become temporal migrants.

Each individual possessed an array of 100 unlinked diallelic loci that are at Hardy–Weinberg equilibrium at the start of the simulation. For each deme and generation, the model calculated allelic frequencies for each locus. Assuming panmixia, for each deme the model obtained genotypes for individuals of a given generation sampling from a binomial distribution based on allelic frequencies from the previous generation. At generation 100, the model generated an input file for the program Arlequin v.3.5.1.2 (Excoffier and Lischer 2010) including individual genotypes for each deme. We estimated gene diversity (*H*_S_) and genetic differentiation between demes and among individuals within demes with a hierarchical analysis of molecular variance (AMOVA). *F*_ST_ statistics (Weir and Cockerham 1984) and their significance were calculated from 1000 permutations. We computed mean *F*_ST_ values for genetic differentiation between demes and the proportion of significant *P*-values for each *F*_ST_ value using the 100 replicates for each simulation scenario including all migration rates and migration event probabilities. We used Euclidean distances between centroids of DAPC to estimate genetic distance between demes, as described above for empirical data.

The model was implemented in R (R Development Core Team 2011) and the script is available in the Supplementary Material (see below).

## Results

### Empirical estimates

The demographic survey conducted on four *E. mediohispanicum* sites between July 2010 and June 2012 allowed the estimation of temporal migration rates that varied from a low of 15 % to a high of 67 % (Table 1). On average, mortality rate of vegetative individuals was 36.52 ± 9.76 % in 2010–11 and 53.95 ± 13.20 % in 2011–12 (Table 1).

**Table 1.** Demographic field data for each transition and sampling site including the total number of tagged *E. mediohispanicum* individuals. We show re-sampled individuals the next year (re-sampled), the number of tagged vegetative rosettes that died during the study year (dead), the number of tagged vegetative rosettes that remained as vegetative (vegetative), the number of tagged vegetative rosettes that became reproductive (reproductive), the estimated mortality rate and the estimated temporal migration rate (the ratio between vegetative and re-sampled individuals).

Transition	Site code	Re-sampled	Dead	Vegetative	Reproductive	Mortality (%)	Temporal migrants (%)
2010–11	Em01	103	42	33	28	40.78	54.10
	Em08	37	16	9	12	43.24	42.86
	Em23	104	23	12	69	22.12	14.81
	Em25	95	37	11	47	38.95	18.97
2011–12	Em01	33	20	5	8	60.61	38.46
	Em08	56	35	14	7	62.50	66.67
	Em23	80	31	10	39	38.75	20.41
	Em25	1	–	–	1	–	–

On average, the mean number of alleles per locus was 9.53 ± 0.59 (range *n*_a_ = 7.89–10.56 alleles per locus; Table 2), observed heterozygosity was 0.57 ± 0.04 (range *H*_O_ = 0.48–0.65; Table 2) and mean expected heterozygosity was 0.75 ± 0.02 (range *H*_S_ = 0.72–0.78; Table 2).

**Table 2.** Genetic diversity data including vegetative and reproductive groups of *E. mediohispanicum* individuals (total *N* = 687) for each year and sampling site. Each developmental state for a given year and sampling site represents a different deme. The genetic parameters include the mean number of alleles per marker (*n*_a_), observed heterozygosity (*H*_O_) and expected mean heterozygosity (*H*_S_). Standard deviations (±SDs) for each parameter are also given.

		2010		2011		2012	
Parameter	Site code	Vegetative	Reproductive	Vegetative	Reproductive	Vegetative	Reproductive
*n*_a_	Em01	8.67 ± 4.87	10.56 ± 5.60	9.33 ± 4.78	9.89 ± 5.53	8.56 ± 4.07	9.33 ± 4.33
	Em08	9.22 ± 3.77	9.89 ± 4.54	9.67 ± 5.07	9.22 ± 4.52	7.89 ± 3.59	9.00 ± 3.91
	Em23	9.56 ± 4.13	9.56 ± 4.59	9.44 ± 4.16	9.56 ± 4.33	10.00 ± 4.87	10.44 ± 5.27
	Em25	9.11 ± 4.08	9.11 ± 4.29	9.67 ± 4.30	9.22 ± 4.63	–	9.89 ± 3.95
*H*_O_	Em01	0.51 ± 0.18	0.61 ± 0.18	0.62 ± 0.15	0.65 ± 0.14	0.55 ± 0.15	0.59 ± 0.13
	Em08	0.57 ± 0.24	0.57 ± 0.24	0.57 ± 0.11	0.57 ± 0.18	0.55 ± 0.17	0.52 ± 0.15
	Em23	0.57 ± 0.23	0.54 ± 0.21	0.59 ± 0.17	0.59 ± 0.20	0.53 ± 0.16	0.48 ± 0.14
	Em25	0.61 ± 0.08	0.57 ± 0.07	0.59 ± 0.06	0.60 ± 0.06	–	0.58 ± 0.13
*H*_S_	Em01	0.75 ± 0.14	0.78 ± 0.18	0.73 ± 0.14	0.76 ± 0.13	0.72 ± 0.15	0.75 ± 0.16
	Em08	0.76 ± 0.14	0.74 ± 0.13	0.74 ± 0.18	0.72 ± 0.18	0.73 ± 0.13	0.73 ± 0.16
	Em23	0.76 ± 0.12	0.76 ± 0.14	0.74 ± 0.16	0.75 ± 0.14	0.76 ± 0.12	0.78 ± 0.13
	Em25	0.76 ± 0.13	0.77 ± 0.12	0.72 ± 0.15	0.74 ± 0.15	–	0.77 ± 0.11

Hierarchical analysis of molecular variance indicated that sites were not significantly differentiated (Table 3). In contrast, year and developmental stages were significantly differentiated although accounting for a low amount of genetic variance (Table 3). Genetic differentiation was also more important between stages than among geographic locations in 2 out of the 3 years studied (Table 4). In both cases, almost all genetic variance was among individuals (Tables 3 and 4). Based on these results, it is not surprising that STRUCTURE was not able to find genetic structure in any set of *E. mediohispanicum* individuals tested in this study **[see****Supporting Information—Appendix S1**, **Fig. S2****]**.

**Table 3.** Hierarchical analysis of molecular variance components and hierarchical *F*-statistics over all loci, including four *E. mediohispanicum* sites (‘Total’) sampled during 3 years (‘Year’), each showing two groups of plants that differ in developmental stage (vegetative and reproductive, ‘Stage’) representing two demes. For each level (including individuals within stage) we show the estimated *F*-statistic and the confidence interval (CI) after 1000 bootstrap replicates. *F*-statistics are significant if CI does not include zero (represented in bold).

Levels	*F*-statistic	CI (2.5–97.5 %)
Population/Total	0.0008	−0.0018–0.0031
Year/Total	**0.0086**	0.0047–0.0125
Stage/Total	**0.0114**	0.0077–0.0148
Individual/Total	**0.2131**	0.1178–0.3366

**Table 4.** Hierarchical analysis of molecular variance including four *E. mediohispanicum* sites. Each site is composed of two groups of plants that differ in developmental stage (vegetative and reproductive) representing two demes. Degrees of freedom (df), percentage of variation of each hierarchical level and their respective fixation indexes with their *P*-values are given. (*) Population Em25 was excluded for 2012 estimation due to the lack of vegetative individuals.

	2010				2011				2012 (*)			
Site code	df	Variation (%)	Fixation index	*P*-value	df	Variation (%)	Fixation index	*P*-value	df	Variation (%)	Fixation index	*P*-value
Among sites	3	0.21	0.0021	0.1896	3	0.42	0.0042	0.06256	2	0.96	0.00959	<0.0001
Between stages (sites)	4	0.89	0.0089	0.042	4	0.77	0.0078	0.02727	3	0.31	0.00312	0.72434
Within stages	440	98.89	0.011	<0.0001	450	98.8	0.012	<0.0001	348	98.73	0.01269	0.00391
Total	447				457				353			

**Figure 3.**
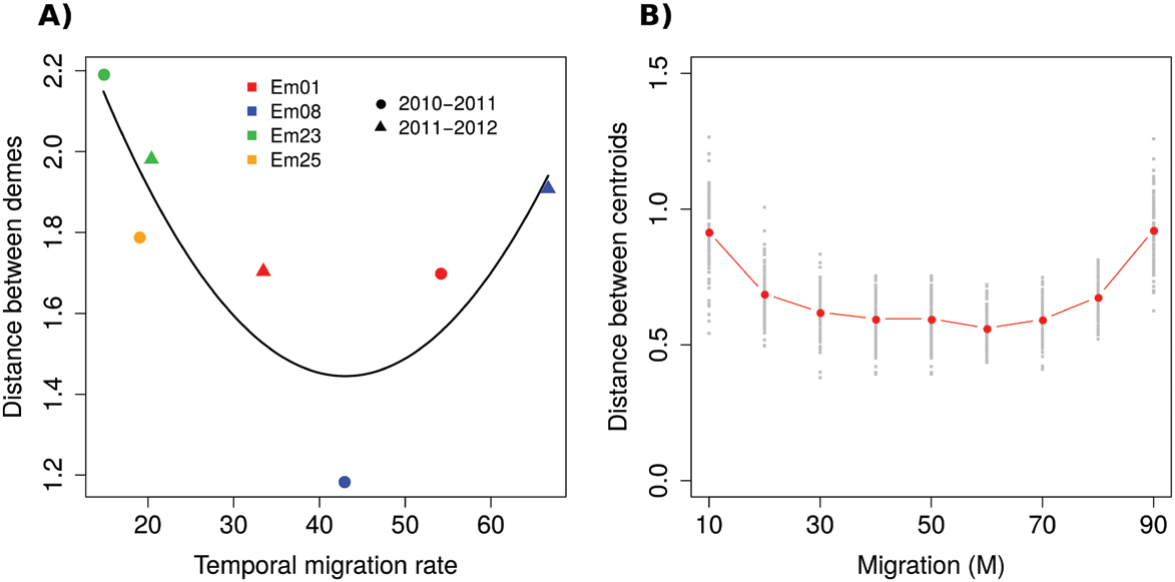
(A) Genetic distance between demes within a population and year estimated from natural *E. mediohispanicum* populations as a function of the observed temporal migration rates. (B) The same relationship but estimated using data simulated by our model.

In contrast, genetic differences between demes estimated as distances between centroids obtained from DAPC did yield interpretable results when compared to field data. As hypothesized, genetic distances between demes within a population and year depended on the observed temporal migration rate for that particular population. However, the relationship between genetic differences between demes and temporal migration rates was not entirely as expected. We found a turning point in the relationship between the variables: decreasing genetic distances with low–moderate temporal migration rates (up to temporal migration rates of 40–50 %) and increasing genetic distances with high temporal migration rates above 50 % (Fig. 3A).

### Model simulations

**Figure 4.**
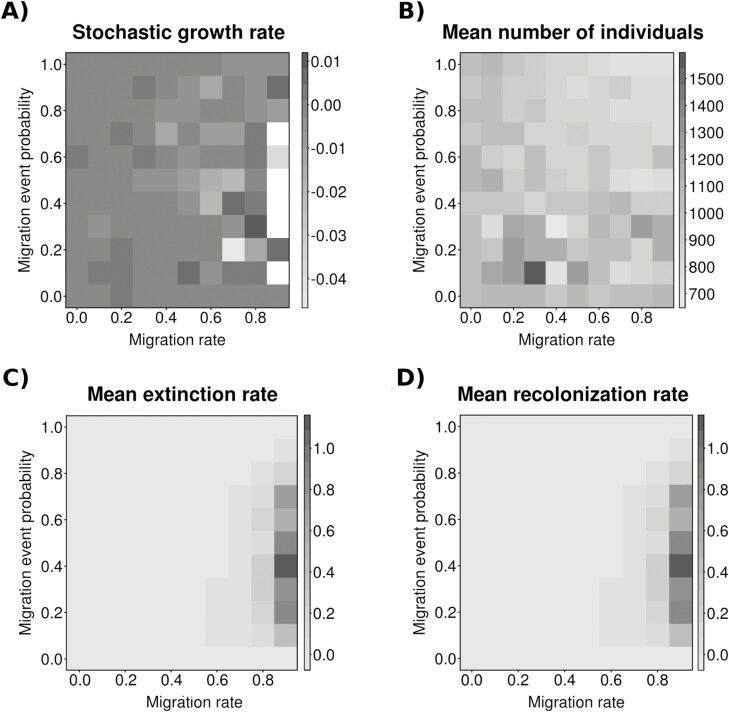
Demographic behaviour of the model across all migration rate and migration event probability scenarios: (A) stochastic deme growth rate, (B) mean number of individuals per deme, (C) mean extinction rates per deme and (D) mean recolonization rates per deme. For the sake of clarity, the results are based on data from one of the two demes because the complementary deme exhibited the same pattern (results not shown). Migration rates vary from 0 to 0.9 and migration event probabilities from 0 to 1.0 with 0.1 intervals. Results were obtained averaging 100 simulations per scenario.

**Figure 5.**
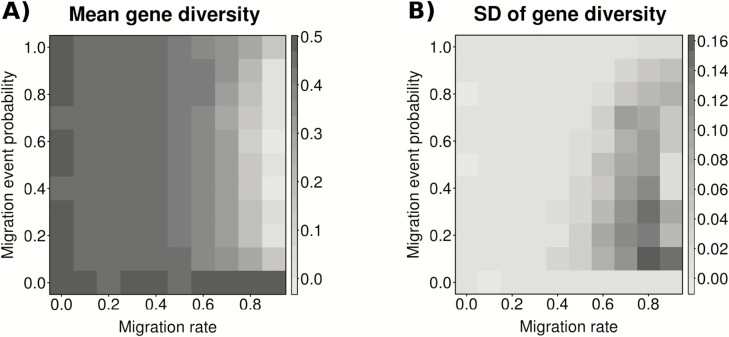
Genetic behaviour of the model across all migration rate and migration event probability scenarios: (A) mean gene diversity between demes (*H*_S_), and (B) standard deviation of mean *H*_S_. Migration rates vary from 0 to 0.9 and migration event probabilities from 0 to 1.0 with 0.1 intervals. Results were obtained averaging 100 simulations per scenario.

The strongest effect of temporal migration on the simulated populations was observed for large migration rates and intermediate migration event probabilities. These scenarios maximized the probability of deme extinction and recolonization, minimized stochastic growth rate values (Fig. 4), and affected genetic diversity by reducing deme mean values and increasing standard deviation values (Fig. 5). These patterns were probably due to the higher stochastic fluctuations observed at high migration rate which affects, in turns, to extinction/recolonization dynamics and diversity **[see****Supporting Information—Fig. S1**, **Appendix S1****]**. In contrast, the mean number of individuals per deme reached the minimum values for higher migration event probabilities (Fig. 4).

**Figure 6.**
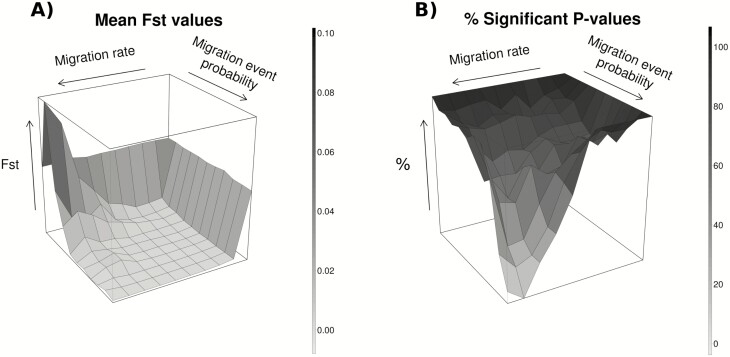
Genetic structure between demes across all migration rate and migration event probability scenarios given by (A) *F*_ST_ values and (B) their respective percentage of significant *P*-values. Migration rates vary from 0 to 0.9 and migration event probabilities from 0 to 1.0 with 0.1 intervals. Results were obtained averaging 100 simulations per scenario.

Temporal genetic differentiation between demes estimated using *F*_ST_ values was low (around 0.05) but significant in all the scenarios with no migration and the proportion of significant *P*-values decreased as migration rate and migration event probability increased (Fig. 6). The largest *F*_ST_ value (0.10) in our simulation was obtained for higher migration rates and low migration event probabilities.

Interestingly, model simulations mimicked the results obtained with empirical data. In particular, genetic distances between simulated demes also showed a U-shaped relationship between genetic differences between demes and temporal migration rates, with decreasing genetic distances with increasing temporal migration rates, and increasing genetic distances with higher temporal migration rate (Fig. 3B).

## Discussion

Theoretically, populations of biennial organisms should be composed of individuals with homogenous developmental stages, either vegetative or reproductive at a given year. However, vegetative and reproductive individuals co-occur in natural populations of biennials (Gross 1981; Kelly 1985; De Jong and Klinkhamer 1988; Suzuki *et al.* 2003; Petrů 2005; Valverde *et al.* 2016). This means that, whatever the reasons, temporal asynchrony of at least 1 year in the completion of the life cycle must have occurred between groups of individuals. Annual asynchrony in reproduction among individuals within populations, defining demes in our biennial plant *E. mediohispanicum*, leads to temporal genetic structure of populations. Examples of the consequences of such temporal genetic structure in biennial plant populations are scarce in the literature (Wells and Wells 1980; Valverde *et al.* 2016). Despite interesting questions are still open (such as whether the propensity to flower in year 2 or year 3 is heritable and whether flowering asynchrony of individuals is a heritable trait and whether temporal migrant and non-migrant individuals differ in size and timing of flowering), our study provided double evidence, empirical and simulated, for the existence of temporal structure in populations in the biennial *E. mediohispanicum*.

We expected genetic differences between demes within *E. mediohispanicum* populations to diminish with increasing temporal migration rates between them, because of the genetic homogenization caused by migration (Slatkin 1985, 1987; Charlesworth *et al.* 2003; Holderegger *et al.* 2010). The combination of empirical estimates of temporal migration rates from natural populations with estimates of genetic structure obtained with DAPC showed that our hypothesis was partially correct, but with an important particularity. As expected, the relationship between temporal migration rates and genetic differences between demes within populations was negative as long as temporal migration rates were below 50 %. However, when temporal migration rates were above 50 %, genetic differences between demes went up again, eventually depicting a U-shape relationship between temporal migration rates and genetic differences between demes within populations (Fig. 3A). Interestingly, the simulations performed also yielded the same U-shape relationship between the same two parameters (Fig. 3B), providing additional support to our finding and revealing how a simple model is able to capture the genetic effects of (temporal) migration on populations. As far as we know, this is the first work reporting this U-shape dependency between genetic differentiation and temporal migration. Although the negative relationship of the U-shape function between temporal migration rates and genetic differences among demes within populations is easy to understand, the positive relationship between the same traits emerging above 50 % of temporal migration rates deserves further attention (but **see****Supporting Information—Appendix S2**).

Temporal migration might represent an advantage for a biennial because populations are made of temporally distributed subpopulations co-occurring in the same location. Two viable subpopulations exchanging genes over time should positively contribute to at least the maintenance of genetic variation of the entire system (Gillespie 1975; Star and Spencer 2013). Our results supported this expectation in two different ways. First, field data showed that reproductive and vegetative groups of individuals from different populations exhibited high gene diversity values with little differences between them (Table 2). Second, simulations clearly indicated that mean gene diversity between demes fluctuated little around a high value between low and medium temporal migration rates (Fig. 3A). In contrast, temporal migration could have some adverse consequences for gene diversity at high migration rates because intense migration in biennials represents an important loss of individuals in the donor population. In fact, migrants are established vegetative rosettes, which are the survivors of massive mortality events commonly recorded among seedlings and juveniles of annual and short-lived plants (Quintana-Ascencio *et al.* 2003; Menges and Quintana-Ascencio 2004; Picó and Retana 2008; Montesinos *et al.* 2009; Picó 2012). Thus, sharp declines in population size due to temporal migrants eventually have detrimental effects on population performance and viability. Our simulations depicted this pattern showing lower growth rates and higher extinction and recolonization dynamics (Fig. 4), which in turn led to decreasing gene diversity at high migration rate scenarios (Fig. 5A). We believe that such dramatic changes in deme population size due to high temporal migration rates, which resemble the effects of demographic stochasticity on population fate (Menges 1991; Fischer and Matthies 1998; Quintana-Ascencio *et al.* 1998; Matthies *et al.* 2004; Menges and Quintana-Ascencio 2004), accounted for the change of trend between temporal migration rates and genetic differences among demes within populations at high migration rates.

The four *E. mediohispanicum* populations were not genetically structured. In addition, the analysis of the distribution of genetic diversity within and among populations, albeit significant, indicated that the genetic differentiation among the four *E. mediohispanicum* populations was very low. We believe that the highly diverse pollinator community visiting *E. mediohispanicum* plants (Gómez *et al.* 2007), which represents high effective gene flow rates among populations, accounts for the lack of genetic structure across the entire system. Furthermore, it is well known that reproductive *E. mediohispanicum* in rainy years becomes ubiquitous across the whole area including the four study sites (authors’ personal observation). Hence, gene flow and genetic homogenization among sites can rapidly increase in that particular area, erasing population structure at the population level. Interestingly, our analyses only detected interpretable signals of genetic structure between demes within populations when using DAPC rather than the Bayesian clustering method STRUCTURE, probably because the former has the power to unravel complex population subdivisions (Jombart *et al.* 2010) due to the mathematical properties of the Discriminant Analysis (which maximizes the differences between groups while minimizes variation within groups).

In summary, our empirical and modeling approaches showed consistent patterns between genetic differences between demes within populations and temporal migration rates in *E. mediohispanicum*. We believe that temporal genetic structure could emerge with more intensity in systems with lower effective gene flow. Our study shed light on the complexity of population genetic features of some biological systems, such as biennial plants, in which the extent of temporal migration rates between demes within populations determines temporal genetic structure through migration or demographic stochasticity.

## Supporting Information

The following additional information is available in the online version of this article—

**Table S1**. Schematic representation of the model procedure.

**Appendix S1**. Model simulations.

**Appendix S2**. Interpretation of the U-shape relationship.

**Figure S1**. Representative simulations pinpointing population dynamics for central (0.5) and extreme values of migration rate (0.0 and 0.9) and migration event probabilities (0.0 and 1.0).

**Figure S2.** Summary of STRUCTURE analysis results.

plaa037_suppl_Supplementary_Material

plaa037_suppl_Supplementary_Data

## Data Availability

Data available in the Supplementary Material.
