# Supplementary material for: Temporal migration rates affect the genetic structure of populations in the biennial Erysimum mediohispanicum with reproductive asynchrony
Source: AoB Plants. 2020 Jul 25;12(4):plaa037. doi: 10.1093/aobpla/plaa037 (PMC7454028; doi:10.1093/aobpla/plaa037)
Supplement: plaa037_suppl_Supplementary_Material [file plaa037_suppl_supplementary_material.pdf]

SUPPORTING INFORMATION

Temporal migration rates affect the genetic structure of populations in the biennial *Erysimum mediohispanicum* with reproductive asynchrony

Table S1 Schematic representation of the model procedure. In a biennial system, at each generation *t*, deme *d1* is found in year *y* whereas deme *d2* co-occur in year *z*. Hence, year counter doubles generation counter. As a result, generations of deme *d1* and *d2* take places in odd and even years, respectively (black arrows). Migration rates m_{12} or m_{21}, from deme *d1* to deme *d2* and vice-versa, respectively, represent the proportion of non-reproductive plants that do not flower after two years of vegetative growth (red arrows). Migration rates will take values of 0, m_{12} or m_{21} according to the defined migration event probability. All 0 values represent no migration in that year and deme. The directionality of migration events between demes determine immigration functions given in Eq. 8 and 9.

Generations	Years		Migration rates	
t	y	z	m_{12}	m_{21}
1	1	2	0	m_{21}
2	3	4	0	0
3	5	6	0	0
4	7	8	m_{12}	0
5	9	10	0	0
6	11	12	0	m_{21}
7	13	14	0	0
8	15	16	m_{12}	0
9	17	18	0	0
10	19	20	0	0
...	...	...	...	...
99	197	198	m_{12}	m_{21}
100	199	200	0	0

Appendix 1 to "Temporal migration rates affect the genetic structure of populations in the biennial *Erysimum mediohispanicum* with reproductive asynchrony"

Model simulations

The demographic model exhibited a purely stochastic behavior across all migration rate and migration event probability scenarios as shown by stochastic deme growth rate (Fig. 4A). The baseline simulation with no migration events yielded a fairly constant stochastic growth rate (Fig. 4A). For low migration rates independently of migration event probabilities, demes fluctuated around stability (Fig. 4A). In contrast, stochastic deme growth rates turned out to be more variable as migration rates increased at intermediate values of migration event probabilities (Fig. 4A). The mean number of individuals per deme decreased with increasing migration rate and mainly with increasing migration event probability (Fig. 4B). This pattern was explained by higher extinction rates at scenarios with high migration rate and intermediate migration event probabilities (Fig. 4C). In this simulated system, extinctions and recolonizations were correlated (Fig. 4C and 4D). In fact, an extinction event in one deme implied an increase in deme size in the other complementary deme or a recolonization event if that deme became extinct. However, recolonizations did not necessarily take place right after an extinction event, but after a migration event. Both extinctions and recolonizations reached their highest values when migration rates were high and migration event probabilities were intermediate (Fig. 4C and 4D).

On average, deme gene diversity (H_S) reached the highest values when migration rates were lower than 0.4 across all migration event probability scenarios, except when migration event probability was zero (Fig. 5A). The no temporal migration scenario simulated a strict biennial habit. In this case, H_S did not change across scenarios because demes did not

exchange individuals. When considering patterns of variation in H_S, we found that standard deviation of H_S increased with increasing migration rates (Fig. 5B). For high migration rates, migration event probability also had an effect on variation in H_S decreasing this variation with increasing migration event probabilities (Fig. 5B). Higher stochastic fluctuations and more pronounced extinction/recolonization dynamics at high migration rate scenarios (Fig. S1) accounted for the patterns of variation found for genetic diversity and genetic structure yielded by the model.

Given the above-described demographic and genetic behavior of the model, temporal genetic structure between demes indicated by F_{ST} values did not change for scenarios with no migration (Fig. 6A). In such scenarios, F_{ST} values smoothly fluctuated around 0.05, differences between demes being significant in all simulations (Fig. 6B). The proportion of significant F_{ST} values sharply decreased with high migration rates and high migration event probabilities (Fig. 6B). In fact, only 11 out of 110 possible scenarios combining migration rates and migration event probabilities showed a proportion of significant F_{ST} values lower than 50%. F_{ST} values were low for most migration scenarios (on average F_{ST} = 0.003 ± 0.0004 for migration rates between 0.1 and 0.5) and increased when migration rates were above 0.5 (on average F_{ST} = 0.012 ± 0.003) and migration event probabilities remained low (Fig. 6A).

Figure S1 Representative simulations pinpointing population dynamics for central (0.5) and extreme values of migration rate (0.0 and 0.9) and migration event probabilities (0.0 and 1.0). Blue and green lines show variation in population size for each deme within the population along the 100 simulated generations. For no migration rates or no migration event probabilities (the three top panels), the population dynamics exhibited a purely stochastic behavior. Central values of migration event probabilities with migration rate higher than zero (the three central panels) show fluctuating dynamics with extinction and recolonization events that in turn increase their frequency with increasing migration rates. Finally, for a migration event probability of 1 (the three bottom panels), population size reaches equilibrium and the number of generations to reach equilibrium is shorter as migration rates are higher.

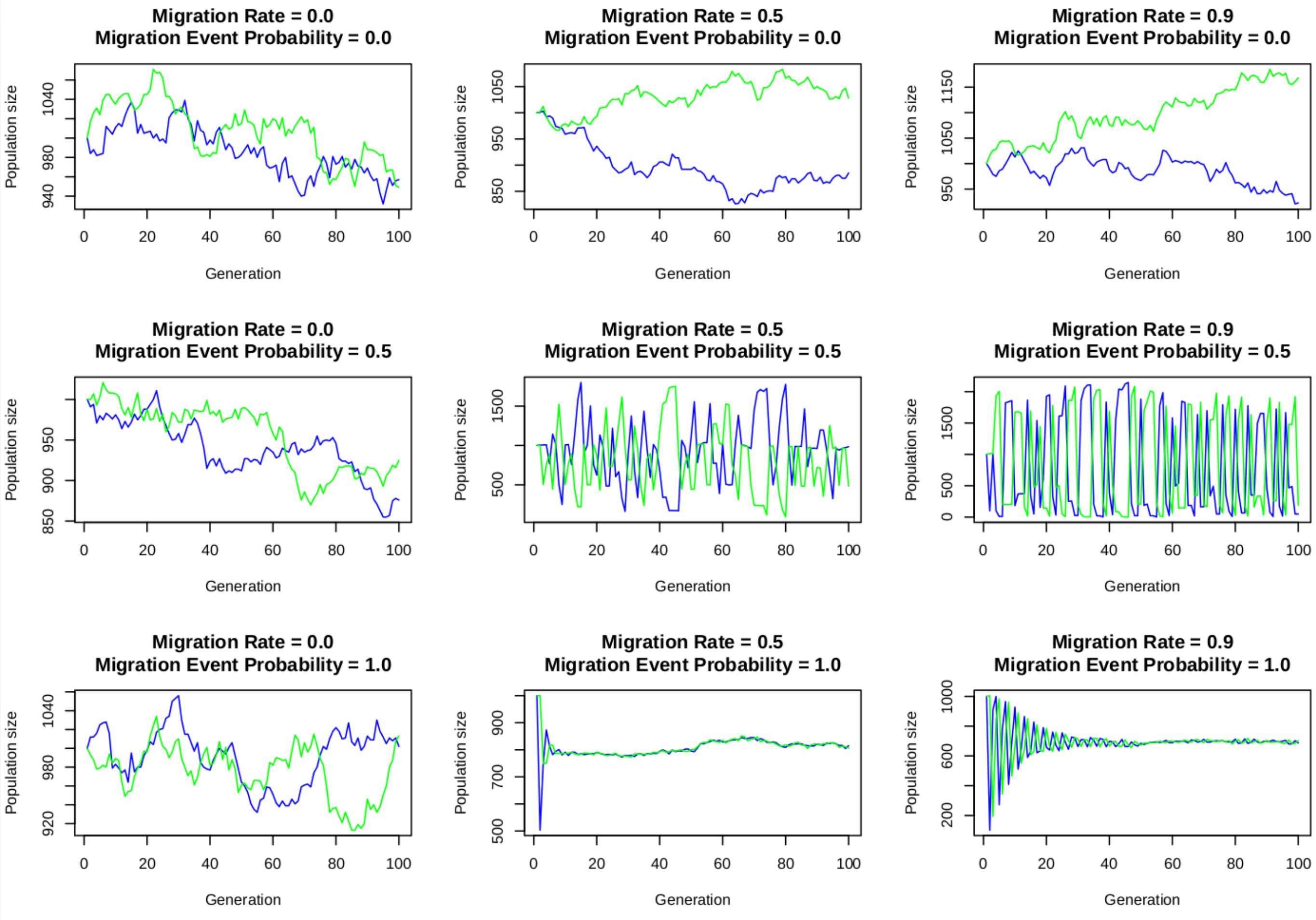

Figure S2 Summary of STRUCTURE analysis results. (A) Probabilities for each *K* estimated for the entire set of 678 individuals using the Evanno method. This result suggests that the most likely value for K is 2. (B) Ancestry membership proportions for individuals using K=2 (values for the 15 replicates are shown). (C) Ancestry membership proportions for individuals using K=10 (values for the 15 replicates are shown). (D) Probabilities for each *K* estimated for the subset of 181 individuals from population Em01. This result suggests that the most likely value for K are 3 and 2. (E) Ancestry membership proportions for individuals using K=2 (values for the 15 replicates are shown). (F) Ancestry membership proportions for individuals using K=3 (values for the 15 replicates are shown). Patterns shown in D-F are identical for all the populations analysed in this study.

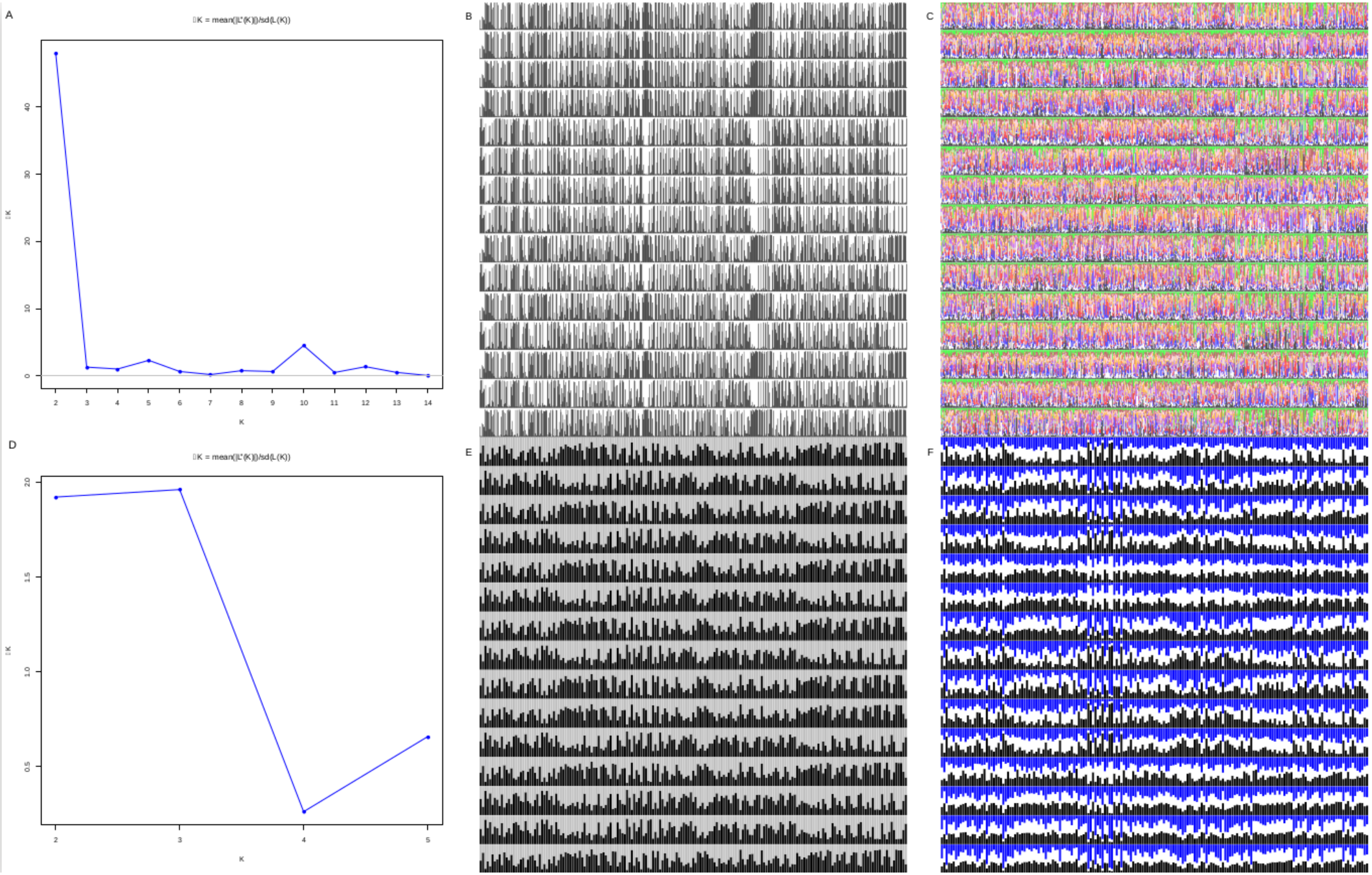

Appendix 2 to "Temporal migration rates affect the genetic structure of populations in the biennial *Erysimum mediohispanicum* with reproductive asynchrony"

Both, empirical data and modeling results found a U-shape relationship between genetic structure and temporal migration rates (Fig. 3). Thus, the minimum genetic distance between demes is found for migration rates around 0.5 (m=0.5) and symmetrical negative and positive relationships are found for m values lower and higher than 0.5, respectively. Despite the negative trend is intuitively explained (the larger the frequency of migrants, the lower the genetic distance between demes), understanding the positive trend requires a deeper insight into the temporal evolutionary dynamics.

Because our model contains two demes and every temporal migrant leaving one deme goes to the other, genetic differentiation patterns produced by the migration rate m are identical to those produced by the complementary migration rate (that is (1-m) or (m-1)). In other words, if 20% of deme 1 migrates, these individuals will reproduce with 80% of deme 2 (because 20% of deme 2 will also migrate). If 80% of deme 1 migrates, these individuals will reproduce with 20% of deme 2 (because 80% of deme 2 will also migrate). In both cases, reproduction occur between 80% of one deme and 20% of the other.

The following picture represents the genetic structure produced between two demes by migration rates 0.2 (top panels) and 0.8 (bottom panels). Four reproductive events (from t=1 to t=4) are shown and reproductive individuals at a given time are represented within the blue polygon. The starting point in both scenarios is a geographic population composed of 10 individuals from deme 1 (black circles) and 10 individuals from deme 2 (white circles).

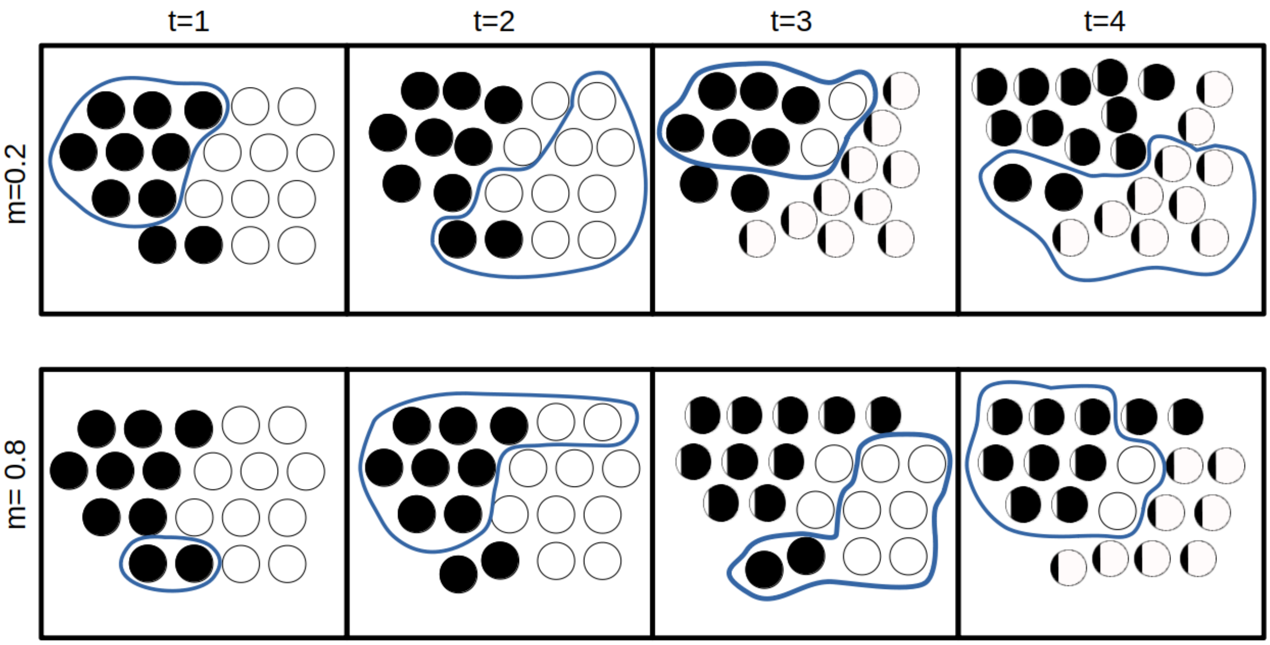

If m=0.2 at time 1 (top left upper panel), only 80% of the deme 1 reproduces (the 8 individuals within the blue polygon) whereas the remaining 20% of the black individuals remain as vegetative. If m=0.8 at time 1 (bottom left upper panel), only 20% of the deme reproduces (2 individuals, blue polygon). In both cases, the number of descendant in t=2 is equal to the number of reproductive individuals in t=1 (8 and 2, respectively), thus maintaining population size constant in time. During t=2, a proportion (20% or 80%) of deme 2 is reproducing together with temporal migrant from deme 1 (blue polygon). The same is true for t=3 and t=4. The offspring produced after reproduction in t=2 is represented with combinations of black and white within individual in time 3. Patterns observed for m=0.2 and m=0.8, are “symmetrical” from t=2 forward. Thus, for t=2, reproduction involves two individuals from one deme and eight from the other whereas for t=4 reproduction involves two individuals from one deme and eight “mixed” individuals. The main difference between m=0.2 and m=0.8 at time t is the (complementary) relative contribution of demes to the

reproductive event occurring during this time. However, the genetic distance between demes is expected to be similar in both cases, resulting in the observed U-shape relationship between genetic differentiation and temporal migration rate.

red	15	A1R40	1	197200	132161	166181	132144	148148	168169	168171	192192	184184
red	15	A1R41	1	197197	135161	175181	132132	160160	168168	186168	180180	170172
red	15	A1R42	1	0	135135	0	140160	156160	172176	168186	180180	176176
red	15	A1R43	1	197200	135176	175178	138140	148158	0	171171	192192	184192
red	15	A1R44	1	0	117132	166178	132132	160178	166166	169171	180180	178178
red	15	A1R45	1	197200	135173	175183	132138	176176	0	171171	192192	170172
red	15	A1R46	1	200200	126129	172175	132138	166178	0	171186	180192	170172
red	15	A1R47	1	197206	129179	166175	138142	156156	168168	171186	192192	170172
red	15	A1R48	1	197197	117173	166181	140140	148158	158158	168186	192192	170174
red	15	A1R49	1	200206	135135	166166	138140	148158	166174	171171	180192	0
red	15	A1R50	1	197206	117176	166166	138138	160176	158158	171186	192192	172176
red	15	A1R51	1	197197	161182	181181	140142	160176	158158	171186	0	184192
red	15	A1R52	1	200206	132132	166175	138150	148156	174188	171171	180192	170170
red	15	A1R53	1	197200	129132	166175	132138	152160	174174	171171	180180	170170
red	15	A1R54	1	200206	141152	166166	132138	172178	0	171171	180192	172178
red	15	A1R55	1	197200	0	175181	132156	152178	164174	168171	180192	168190
red	15	A1R56	1	197197	120152	178181	132132	160160	158172	171171	0	176184
red	15	A1R57	1	197197	152167	166166	134136	158160	158158	171171	192192	172182
red	15	A1R58	1	206206	144152	166175	138140	160178	158158	171171	180192	174192
red	15	A1R59	1	197200	152161	166166	140146	148162	148148	171171	192192	178178
red	15	A1R60	1	200203	129132	178181	132138	152152	152152	168171	180180	174174
red	15	A1R61	1	200200	132158	166175	138138	160160	178188	171171	180192	174184
red	15	A1R62	1	200200	129132	166181	132142	150178	160160	168177	180192	172174
red	15	A1R63	1	197206	150176	166175	138156	162178	158158	171177	180192	176192
red	15	A1R64	1	197197	152152	181181	132140	160178	172172	171186	180180	172182
red	15	A1R65	1	0	161173	172175	138140	148160	158158	168168	180192	172192
red	15	A1R66	1	197203	135135	166181	138158	150174	180180	168186	180192	172188
red	15	A1R67	1	188197	120164	175175	132138	0	178178	171171	180192	170196
red	15	A1R68	1	197206	144144	178181	136138	160178	166166	171171	0	178181
red	15	A1R69	1	197197	155173	166175	132138	148172	158174	168171	0	170172
blue	16	A1V40	2	197206	135152	166181	138138	154154	160168	186186	180180	170184
blue	16	A1V41	2	197200	164170	166181	132156	148152	160160	168171	180180	170174
blue	16	A1V42	2	197206	135135	166178	132156	160178	166166	171171	180192	170172
blue	16	A1V43	2	200200	129164	181181	138138	148158	172172	171171	180192	0
blue	16	A1V44	2	197200	138164	178181	132132	148148	158176	168186	180192	172174
blue	16	A1V45	2	197197	117117	166175	132136	160160	158158	171171	180180	184184
blue	16	A1V46	2	197200	129176	181181	132138	148148	160160	168171	180192	174180
blue	16	A1V48	2	206206	129164	175175	132138	160162	158178	171171	180192	172172
blue	16	A1V49	2	197197	129132	175175	138146	148158	0	168171	180192	170170
blue	16	A1V50	2	197200	132176	175181	138138	162162	166166	171171	180192	170170
blue	16	A1V51	2	197197	132132	166175	138138	148162	158176	168186	180180	170176
blue	16	A1V52	2	197206	147158	166181	134138	158160	168168	171171	0	0
blue	16	A1V53	2	197197	132132	175181	138138	152162	168168	0	0	0
blue	16	A1V54	2	197200	117141	178181	138138	148156	158158	168186	180192	180180
blue	16	A1V55	2	200206	150191	175181	156160	150160	160166	171171	180192	168174
blue	16	A1V56	2	0	117132	178181	138138	148158	158158	171171	192192	174175
blue	16	A1V57	2	0	129155	0	132132	152162	158158	171186	180180	172180
blue	16	A1V58	2	200200	132158	181181	132138	178178	158158	168171	180192	174174
blue	16	A1V59	2	200200	164164	175181	132142	160178	158174	171171	192192	170174
blue	16	A1V60	2	194200	132138	178181	132132	162162	168168	171171	180180	174182
blue	16	A1V61	2	197197	120158	175178	132138	162162	0	171171	183183	176178
blue	16	A1V62	2	197206	0	166181	138138	148152	0	171171	180180	170170
blue	16	A1V63	2	197200	132138	166175	132138	148162	160160	171171	180192	192192
blue	16	A1V64	2	197197	132158	166181	132132	148178	160160	171186	180192	176184
blue	16	A1V65	2	197197	132158	175175	138156	160178	166166	186186	180180	0
blue	16	A1V66	2	197197	152164	175175	132138	156156	166166	171171	192192	180186
blue	16	A1V67	2	197197	161161	175181	138138	148176	160160	171177	180192	170174
blue	16	A1V68	2	200200	135167	166166	138138	178178	158158	0	0	174174
blue	16	A1V69	2	200200	147176	178181	132138	158178	172172	171171	180180	176176
blue	17	B1R30	3	197197	135164	166181	132132	148158	162162	177177	180192	170174
blue	17	B1R31	3	197200	120132	175181	132138	148160	158158	168171	180192	168176
blue	17	B1R32	3	197200	117150	166181	134140	148178	158158	168171	180192	0
blue	17	B1R33	3	203203	150170	166183	140142	160178	164176	168186	183183	170188
blue	17	B1R34	3	197197	138164	175181	136138	148148	148148	168171	180180	176192
blue	17	B1R35	3	197197	132158	166181	134138	162178	158158	171171	180192	170172
blue	17	B1R36	3	197200	135164	166183	134140	148164	148158	171171	183183	170180
blue	17	B1R37	3	197206	158188	166178	132132	150150	158168	171171	192192	174192
blue	17	B1R38	3	200206	129161	166175	140158	152156	158174	171171	180180	180192
blue	17	B1R39	3	197203	135182	166183	140158	148174	170180	168186	180192	170180
blue	17	B1R40	3	197200	129138	166183	132132	148164	158158	171171	180180	170170
blue	17	B1R41	3	197197	167191	175175	138140	176176	158170	171171	180192	170172
blue	17	B1R42	3	197197	129191	166166	138140	148168	148168	168186	183183	186188
blue	17	B1R43	3	197203	117129	166175	130140	148168	148148	171186	183183	176176
blue	17	B1R44	3	197197	138173	166181	134138	162178	158158	171171	192192	174174
blue	17	B1R45	3	197197	138161	166175	148156	152158	158158	171171	180183	174174
blue	17	B1R46	3	197197	138161	166175	138138	150178	158158	186186	180192	170170
blue	17	B1R47	3	197212	152152	181183	132162	148164	172172	0	180180	172190
blue	17	B1R48	3	197197	117129	178181	138138	178178	166176	171171	180180	170180
blue	17	B1R49	3	200203	132132	166175	132132	150162	158178	168171	192192	170174
blue	17	B1R50	3	197197	129132	166166	134138	148160	164172	171186	180192	170170
blue	17	B1R51	3	197206	152152	181181	134140	148158	164172	168171	180180	170178
blue	17	B1R52	3	197200	135135	175178	138140	148160	0	171171	192192	174178
blue	17	B1R53	3	197197	135164	166166	138140	148148	174174	171186	192192	170176
blue	17	B1R54	3	197200	129129	166181	138142	148160	158168	186186	180180	174192
blue	17	B1R55	3	197206	150176	166181	140140	152162	158168	171186	180192	170170
blue	17	B1R56	3	197197	132150	166181	132140	148162	156166	168171	180192	172172
blue	17	B1R57	3	200206	144144	166181	132132	162162	166166	171186	180192	170180
blue	17	B1R58	3	197206	152158	166175	140142	148160	166166	171186	180192	174182
blue	17	B1R59	3	197206	129129	166183	132132	150162	170172	168171	180180	170182
blue	17	B1R60	3	188197	141141	166183	132138	178178	174174	171171	180192	172178
red	3	B1V01	4	197200	141141	181181	132140	148162	158158	171171	180192	168170
red	3	B1V02	4	206206	135144	175178	138156	148162	158176	168174	180180	172174
red	3	B1V03	4	197203	129132	0	134138	148162	158158	171171	180192	172172
red	3	B1V04	4	197197	132158	175178	132140	148178	158158	171171	180192	168170
red	3	B1V05	4	197206	129158	166181	140140	162178	158158	171171	180180	192196
red	3	B1V06	4	197197	129132	172175	132138	150162	158158	171186	180180	170174
red	3	B1V07	4	197197	129132	181183	132132	152162	158158	171174	180180	174180
red	3	B1V08	4	197197	117182	175181	134140	150150	158164	168171	180180	174174
red	3	B1V09	4	197200	152182	175181	140140	150162	158158	171171	192192	174180
red	3	B1V10	4	197197	132161	166178	134140	178178	174176	171186	192192	170180

red	3	B1V11	4	197200	135161	166178	136140	150150	176176	168171	180192	170176
red	3	B1V12	4	197197	132138	166166	140142	148162	158178	168171	183183	172174
red	3	B1V13	4	197197	129132	175175	138140	148166	148158	171186	183183	176176
red	3	B1V14	4	197200	129129	175181	0	150162	154164	171171	180192	174188
red	3	B1V15	4	197197	167170	166175	136140	150162	166176	171171	192192	170172
red	3	B1V16	4	197197	158158	178181	138138	150178	0	171171	183192	174186
red	3	B1V17	4	197206	132161	166181	132140	160160	170170	168171	180192	174174
red	3	B1V18	4	197206	132132	178181	138160	154164	170170	171171	180180	174176
red	3	B1V19	4	197197	129176	181181	132132	162178	172172	171171	183183	176178
red	3	B1V20	4	197206	117138	181181	132134	160164	158174	174174	180192	172184
red	3	B1V21	4	197197	129132	166181	134138	148160	158158	0	180180	172172
red	3	B1V22	4	197200	150158	175175	140140	150178	178178	171171	180180	174174
red	3	B1V23	4	197200	141191	166181	140160	150164	158180	168186	192192	174174
red	3	B1V24	4	197197	138161	181183	140142	162162	168170	168186	180192	172172
red	3	B1V25	4	197200	152161	172181	138140	148150	158158	171171	180192	172172
red	3	B1V26	4	197197	129138	166166	138138	148176	148158	171174	180192	172172
red	3	B1V27	4	197200	164167	181181	140140	150150	148158	168171	180192	180192
red	3	B1V28	4	197200	132158	181181	132158	140152	166166	168171	180192	168172
red	3	B1V29	4	194206	129147	166181	140140	148160	170178	171171	180192	170180
red	4	C1R01	5	197197	161161	166175	132138	148176	168168	171171	180192	174176
red	4	C1R02	5	197197	129161	169175	132134	148176	170172	168186	180192	180180
red	4	C1R03	5	188197	117152	175181	132138	156170	162162	171171	192192	170172
red	4	C1R04	5	197197	129167	166183	160160	160176	168168	171171	180180	170178
red	4	C1R05	5	197197	117152	178181	140140	160172	158172	171171	180192	174182
red	4	C1R06	5	197203	144161	178178	138142	148148	168168	171171	180180	172172
red	4	C1R07	5	200200	152161	166181	134138	150150	158168	171186	180192	0
red	4	C1R08	5	194194	158158	166181	132138	148160	168182	171171	180192	170170
red	4	C1R09	5	197197	138150	166175	132140	148158	172172	171171	192192	168168
red	4	C1R10	5	197200	167173	166175	132138	162178	166176	168171	180192	168168
red	4	C1R11	5	197200	164170	166175	138140	160162	158158	171171	180192	172182
red	4	C1R12	5	197200	158158	175178	138144	148176	158188	171186	180192	170186
red	4	C1R13	5	191197	126173	175178	140140	160160	172172	171186	0	170170
red	4	C1R14	5	197200	135135	175181	132138	162162	166166	171186	180180	184192
red	4	C1R15	5	200200	129135	166183	138138	178178	166166	171171	192192	168180
red	4	C1R16	5	197200	132138	175181	138148	0	168168	168186	180192	170174
red	4	C1R17	5	197203	164167	181181	132138	148160	0	168171	180180	174180
red	4	C1R18	5	200206	129167	175183	132138	156158	158176	171186	180180	174180
red	4	C1R19	5	197197	138138	181186	138138	148162	160160	168171	180192	160172
red	4	C1R20	5	197197	170176	181181	132134	158158	160160	168171	180180	174182
red	4	C1R21	5	197200	117170	166181	0	0	158158	186186	180192	170176
red	4	C1R22	5	197197	129167	181181	132132	156156	158168	171171	192192	174174
red	4	C1R23	5	197197	117158	175175	132140	176176	158172	171186	180180	172176
red	4	C1R24	5	197200	129170	166175	132132	148150	160168	171171	180180	170170
red	4	C1R25	5	197200	135135	175175	140140	158178	168168	171171	180192	170174
red	4	C1R26	5	200200	117117	0	132138	148148	168168	168171	180180	172172
red	4	C1R27	5	197206	132135	166181	138140	178176	160174	186186	0	168172
red	4	C1R28	5	197206	129152	166178	132138	162178	0	171171	180180	170174
red	4	C1R29	5	197197	132138	166166	132138	178178	158158	171171	180192	170170
red	4	C1R30	5	197206	158158	166181	138138	176176	158158	168171	180192	170178
red	4	C1R31	5	197197	167170	166178	132144	158160	158172	171171	180195	168180
red	4	C1R32	5	197197	129170	166181	132140	150158	178178	171186	180192	168174
blue	7	C1V01	6	0	129170	166178	132132	160148	168168	0	180192	0
blue	7	C1V02	6	200212	117117	166181	134138	148150	158162	171177	180192	174182
blue	7	C1V03	6	197206	161167	181181	138140	160162	158158	171171	180180	180180
blue	7	C1V04	6	197200	176176	166166	138138	160160	160160	171171	180180	170174
blue	7	C1V05	6	197197	164167	181181	138140	162166	170170	0	0	0
blue	7	C1V06	6	194194	138147	166175	132138	150172	168168	171171	180195	170180
blue	7	C1V07	6	197197	164164	175181	138140	164176	168168	168171	192192	170176
blue	7	C1V08	6	197206	129132	166181	132146	160160	158158	168171	180192	170174
blue	7	C1V09	6	197197	135158	166166	132138	162162	168168	168168	180180	172172
blue	7	C1V10	6	197200	117117	166169	132138	162162	166176	171186	180180	176182
blue	7	C1V11	6	197197	135176	166181	132138	156178	178180	171171	180192	158168
blue	7	C1V12	6	197200	138167	178183	132132	176176	158168	171186	180180	170176
blue	7	C1V13	6	197197	132132	175181	132138	176176	168178	171171	180192	174180
blue	7	C1V14	6	197200	129135	175175	132138	160160	158168	171186	183192	168170
blue	7	C1V15	6	197197	164173	175175	132138	160176	0	171171	180192	170170
blue	7	C1V16	6	197197	164158	166166	138138	150150	166166	168186	180180	170182
blue	7	C1V17	6	197197	152158	166181	138138	148148	158158	171171	180180	172174
blue	7	C1V18	6	197197	135167	178181	138138	148176	158158	171171	180192	174174
blue	7	C1V19	6	197206	158164	166175	138140	148176	158158	171171	180192	174174
blue	7	C1V20	6	197200	135161	169178	138138	148164	178178	171171	180192	176180
blue	7	C1V21	6	197197	129176	175181	132132	160160	162162	168171	180180	170174
blue	7	C1V22	6	197200	117167	178181	138138	148160	158158	171171	180192	174174
blue	7	C1V23	6	197197	0	175181	132140	148162	158158	171186	195195	170172
blue	7	C1V24	6	197197	135150	166178	138140	148164	172172	171186	192192	172172
blue	7	C1V25	6	197203	152161	181181	132138	148176	162176	168171	180180	174184
blue	7	C1V26	6	197197	129129	175175	138140	0	158158	171171	180180	170172
blue	7	C1V27	6	197200	129176	166181	132140	148148	158168	168171	180192	170174
blue	7	C1V28	6	197200	117129	166175	132138	156176	158158	168171	192192	170170
blue	7	C1V29	6	200200	141147	181183	138138	148160	158178	168171	180180	174180
blue	7	C1V30	6	197197	167170	166166	132138	164164	166166	171186	180180	170170

red	15	A8R40	197197	129138	166181	138140	152166	170170	171171	180195	174174
red	15	A8R41	197200	129158	166175	132140	148178	158158	171186	192192	168170
red	15	A8R42	197197	161161	160175	132138	160160	160168	171186	180183	168176
red	15	A8R43	197206	152166	166181	132138	176176	160170	171186	192192	176176
red	15	A8R44	197197	161164	175181	136138	150160	158158	174186	180180	170170
red	15	A8R45	197206	135152	166175	138150	156162	174174	171171	192192	170174
red	15	A8R46	200200	117117	166181	132140	158160	174174	171186	180180	184186
red	15	A8R47	197200	129179	166166	132138	148150	158158	168186	180180	170174
red	15	A8R48	197200	164182	166175	138150	160160	158158	171171	180183	170170
red	15	A8R49	197200	161161	166175	132140	176176	0	171186	180192	180186
red	15	A8R50	197200	158164	178183	140142	148148	188188	171171	180180	182182
red	15	A8R51	197197	161161	175181	134138	160160	170170	171171	180180	172172
red	15	A8R52	197197	158173	166175	142142	148176	178178	171186	180180	170174
red	15	A8R53	197197	117147	166178	138138	148150	176176	168171	180183	176186
red	15	A8R54	197197	117158	166181	136140	148148	170170	168168	180180	174180
red	15	A8R55	0	155155	0	132138	148148	168168	171171	180192	172174
red	15	A8R56	197197	129141	175181	132138	148148	166166	186186	180180	172186
red	15	A8R57	197197	164170	166166	138138	148160	166166	171171	180180	170172
red	15	A8R58	197212	123129	166181	132138	150178	158158	171171	192192	182182
red	15	A8R59	197200	161176	175181	138138	148152	0	168171	180192	176180
red	15	A8R60	197203	117152	166178	138138	156166	158158	171171	180180	170174
red	15	A8R61	197200	132155	175181	140150	158168	188188	171171	0	178178
red	15	A8R62	197206	164167	166175	138140	160160	174174	168186	183195	172178
red	15	A8R63_1	194197	129161	175180	138138	148166	158174	171171	0	170170
red	15	A8R63_2	197200	155176	178181	140140	158160	158158	171177	192192	170188
red	15	A8R64	188197	152176	166181	132138	150160	182182	168171	192195	184188
red	15	A8R66	197200	147161	166166	132136	148160	158158	168171	195195	172180
red	15	A8R67	197197	158176	166166	138138	156178	166166	171171	0	170174
red	15	A8R68	197197	117173	169181	138144	148148	158158	174186	0	168168
red	15	A8R69	197197	152173	169181	138138	0	168168	171186	180192	170174
blue	16	A8V40	200200	158158	181186	138148	176176	158158	168171	192195	168168
blue	16	A8V41	203206	129173	181181	132138	166166	166166	171171	180180	0
blue	16	A8V42	197197	173182	175178	132138	178178	158158	171177	192192	172172
blue	16	A8V43	197197	129170	166181	138146	152156	164164	171186	180180	182186
blue	16	A8V44	197200	0	166181	140150	158160	0	171177	180192	172184
blue	16	A8V45	197200	123167	166175	138138	148150	164164	171171	180180	176184
blue	16	A8V46	197206	158161	178181	132138	148148	172172	171171	180183	170174
blue	16	A8V47	197197	161164	175181	138140	148150	172172	171171	180180	172174
blue	16	A8V49	200206	150167	181181	138138	148150	174174	168186	180180	174174
blue	16	A8V50	197200	161164	175175	136140	148168	158158	168168	180180	168174
blue	16	A8V52	194197	129155	178178	0	158160	158158	168171	192192	174174
blue	16	A8V53	194197	161173	175181	132140	150178	166166	171171	180195	170170
blue	16	A8V54	200206	129129	181181	138138	168178	158158	168171	180180	182182
blue	16	A8V55	0	117129	0	132138	150178	158178	171171	180180	178182
blue	16	A8V56	197206	129141	166166	132160	150150	158158	171186	180192	172178
blue	16	A8V57	197200	158161	166166	132138	148180	158158	171171	0	172178
blue	16	A8V58	197197	129158	175181	132138	178178	158158	171171	180180	174186
blue	16	A8V59	197200	129158	181181	132138	148176	158158	168171	0	170178
blue	16	A8V60	197200	158176	166181	138160	152160	166166	0	180180	168178
blue	16	A8V61	194197	161164	178183	132132	148178	0	171171	180192	168170
blue	16	A8V62	197200	170173	166166	132138	154178	158158	171171	180192	182182
blue	16	A8V63	197200	117158	166166	132138	148150	172172	168171	189192	172186
blue	16	A8V64	197200	161176	166169	132138	148150	158158	171186	180192	174176
blue	16	A8V65	197197	152173	181181	132132	150150	164164	171186	180192	172174
blue	16	A8V66	200203	158182	166183	132132	154178	0	171186	180192	184184
blue	16	A8V67	200206	161161	181181	132138	172174	170170	171171	180192	172180
blue	16	A8V68	197200	164167	166181	138150	148148	158158	168171	180180	168178
blue	16	A8V69	197197	117117	166181	132140	150152	158158	168186	180180	168178
blue	16	A8V70	197197	158117	166175	138138	148160	170170	171171	180180	168170
blue	17	B8R31	197200	132152	166166	150150	160176	158158	171186	192192	180182
blue	17	B8R32	197200	158161	166178	132138	148160	170170	171171	180180	170180
blue	17	B8R33	197200	129158	175175	132132	152152	158158	171174	183192	170170
blue	17	B8R34	200212	161161	166178	132160	158158	162162	171186	192192	174174
blue	17	B8R35	197197	129129	166181	130130	160160	158166	171168	192192	174182
blue	17	B8R36	197197	161167	178178	132138	142142	158158	171174	183192	170170
blue	17	B8R37	197197	132132	175181	132140	156176	158158	171171	180192	174174
blue	17	B8R38	197197	135158	166181	132140	148160	188188	171177	192192	170178
blue	17	B8R39	197200	147147	166181	140142	160176	166172	171171	183192	170176
blue	17	B8R40	197212	173176	172181	134140	148164	158158	171171	180192	178198
blue	17	B8R41	197206	161161	166175	132148	172178	158158	171171	180192	170174
blue	17	B8R42	197197	135164	181181	136136	150164	158158	171171	180180	170180
blue	17	B8R43	197197	120129	181181	132132	158162	170176	171171	192192	170182
blue	17	B8R44	197200	129170	175186	132154	148162	158158	168171	192192	182170
blue	17	B8R45	197197	129144	166181	132140	178178	170170	168171	180180	172180
blue	17	B8R46	197206	135158	166169	132140	150178	168158	171171	180180	168172
blue	17	B8R47	197197	129132	166181	132138	150150	158158	171171	180180	172186
blue	17	B8R48	197197	158164	175178	132140	162162	158158	171186	180192	168174
blue	17	B8R49	197197	164164	172181	132138	178178	188188	171171	180180	168170
blue	17	B8R50	197197	164170	166166	132140	170170	174174	171171	192192	168176
blue	17	B8R51	197200	120182	166175	134158	148148	162162	171171	180180	180180
blue	17	B8R52	197197	117135	166181	138140	162178	170170	171186	180180	172180
blue	17	B8R53	197197	161161	166166	138140	150170	176176	177186	180183	180180
blue	17	B8R54	197197	132161	166181	138140	148148	158176	171186	183183	170174
blue	17	B8R55	197200	158167	175181	132132	150160	176178	171186	183183	170172
blue	17	B8R56	197200	161173	166166	140140	148158	166188	171174	180183	170176
blue	17	B8R57	188206	132158	175181	132132	178178	158170	168171	180180	170174
blue	17	B8R58	197197	129129	181181	140156	156158	158166	171171	180183	174182
blue	17	B8R59	197197	152170	175181	140150	160160	158158	171171	180180	182186
blue	17	B8R60	197197	129170	166175	132140	154158	166166	171186	180180	170172
red	3	B8V01	197206	120132	175181	132140	154160	158166	168171	180180	176176
red	3	B8V02	197200	150161	166181	140150	160180	176178	171171	180192	174174
red	3	B8V03	197200	120150	175181	132138	148158	164174	171171	180180	0

red	3	B8V04	185197	150167	166166	134138	160160	166172	171174	192192	182182
red	3	B8V05	185197	141170	175178	132138	158178	162166	186186	180192	186186
red	3	B8V06	197200	164182	181181	136140	158178	148158	171186	183183	168176
red	3	B8V07	185197	120129	166181	136136	178180	158158	171171	183195	170178
red	3	B8V08	197206	120132	181181	132138	160160	158158	171186	180180	172174
red	3	B8V09	197197	138161	181181	132138	150150	158158	168186	195195	168168
red	3	B8V10	197206	161182	166175	132132	158158	162172	171171	192192	168168
red	3	B8V11	197197	170176	166166	136136	178148	166166	171171	192192	174186
red	3	B8V12	197197	158161	166166	132140	148162	166176	171171	192192	172184
red	3	B8V13	197206	117135	175181	138140	148148	158158	171171	183192	176178
red	3	B8V14	197197	129129	166181	138140	182182	158158	186186	180192	172186
red	3	B8V15	197197	155158	166166	138142	168168	166172	171171	183192	168180
red	3	B8V16	197206	167176	166175	132132	172178	170176	171171	180192	170170
red	3	B8V17	197206	129164	166181	140140	148176	160160	171171	183183	182186
red	3	B8V18	197197	132161	181186	138138	140140	148160	171171	183195	162174
red	3	B8V19	194197	158164	175181	142142	164174	166166	168171	180192	174174
red	3	B8V21	197206	158158	166181	132134	148148	158176	171186	180186	174192
red	3	B8V22	197197	132164	166166	138142	150158	164164	171171	192192	180186
red	3	B8V23	197197	120173	178178	132138	150160	158158	171171	192195	174176
red	3	B8V24	197197	135135	166166	132132	150150	0	171171	180180	172180
red	3	B8V25	197200	135167	166181	132138	160168	166170	186171	180192	172180
red	3	B8V26	197197	129129	166175	132132	158176	170170	171171	180180	172176
red	3	B8V27	197200	170170	166166	132138	160160	158176	171171	192192	172182
red	3	B8V28	197197	129129	166181	140140	164168	158158	171171	192195	174174
red	3	B8V29	197197	155167	166178	132132	148150	158180	171177	180183	168172
red	3	B8V30	197197	161161	166166	132144	156160	158158	168186	180192	172182
red	4	C8R01	200200	129167	175181	140140	164166	158158	171171	180192	174180
red	4	C8R02	200203	135158	166175	132138	160178	158168	168171	192192	0
red	4	C8R03	197203	173167	166166	136140	150152	158166	171171	180192	170174
red	4	C8R04	197197	147167	166183	138152	160160	158166	168186	183183	170170
red	4	C8R05	197197	129176	175175	138138	176176	158178	168171	180180	174176
red	4	C8R06	197200	132161	166166	132132	150160	168168	168186	192192	178186
red	4	C8R07	0	129173	175181	132132	160178	158168	171171	180192	174174
red	4	C8R08	200206	129129	175181	132132	150160	166166	171186	192192	174178
red	4	C8R09	197197	129158	166175	0	158158	166166	171171	192192	168168
red	4	C8R10	197200	132176	175175	148148	148148	158158	171171	180180	174174
red	4	C8R11	197200	129158	175175	138140	150158	158158	168171	180192	174174
red	4	C8R12	197197	129129	166166	132150	158174	174174	171186	180192	180180
red	4	C8R13	197197	161161	175181	138138	158158	162162	171171	0	172186
red	4	C8R14	200206	164164	166178	132140	152162	158168	171171	180192	184184
red	4	C8R15	197197	129147	175181	132140	148178	168180	168171	180192	172172
red	4	C8R16	200206	167170	166175	0	0	158158	171171	192192	172182
red	4	C8R17	0	161164	175175	132132	158158	158174	171171	180180	170172
red	4	C8R18	197197	147167	181186	132138	148148	166188	168171	192192	176180
red	4	C8R19	0	161167	181186	132138	148148	0	171171	192192	176180
red	4	C8R20	197197	161167	181186	132132	148160	158178	171171	192192	174174
red	4	C8R21	0	158161	166181	138140	160162	182182	171171	180180	170170
red	4	C8R22	197197	152152	181186	132132	148160	158178	171171	0	174174
red	4	C8R23	191197	158161	166181	138140	160162	182182	171171	180192	172172
red	4	C8R24	197197	152152	181183	138138	148148	158158	171171	180180	170174
red	4	C8R25	197197	120129	166175	132140	160178	158166	171171	192192	184184
red	4	C8R26	197197	161164	166169	132138	160176	168188	171171	180180	168170
red	4	C8R27	200200	129161	0	136138	160162	176176	171186	192195	168170
red	4	C8R28	200200	132155	175175	132132	160160	158174	168171	183195	170174
red	4	C8R29	197197	135158	166183	138138	160170	180180	171171	180192	172172
red	4	C8R30	197200	129129	166181	132132	148160	156166	171171	180192	160170
red	4	C8R31	200200	161164	175178	138138	160160	176176	171186	180192	160170
red	4	C8R32	197200	158164	166181	132132	160176	158158	171171	180192	172176
blue	7	C8V01	197203	161161	166166	138138	148160	178178	171171	180192	170180
blue	7	C8V02	197203	152173	181186	132152	160162	178178	171186	180180	176182
blue	7	C8V03	197197	147167	175181	132138	176176	178178	171186	192192	172186
blue	7	C8V04	197206	132176	166175	132132	160160	158158	171186	180180	176176
blue	7	C8V05	197206	144167	175181	132132	150150	158168	171171	180180	172172
blue	7	C8V06	200206	152152	166175	132138	150160	166166	168171	180192	182182
blue	7	C8V07	197206	129129	172183	138138	148148	166166	168171	180192	174174
blue	7	C8V08	203206	117138	175175	132150	148158	172172	171186	180180	170170
blue	7	C8V09	197197	129129	175181	138138	148150	158168	171171	192192	184184
blue	7	C8V10	197197	164167	175175	132150	160160	176176	171171	180195	170184
blue	7	C8V11	197197	129152	166178	132132	160160	168168	171171	183183	174174
blue	7	C8V12	197197	132135	175175	132138	160160	158158	171171	180192	168168
blue	7	C8V13	197197	155161	175181	136150	160160	158178	168186	180192	170184
blue	7	C8V14	197197	161164	166175	132132	160160	168168	168171	180192	170170
blue	7	C8V15	197200	138167	169181	132138	158176	158158	171186	186195	174174
blue	7	C8V16	200200	167167	169181	132138	158158	178178	168186	180195	170174
blue	7	C8V17	197200	152173	166175	132140	150150	168168	171177	192195	170170
blue	7	C8V18	197197	117129	178183	138138	178178	168168	168171	192192	170178
blue	7	C8V19	197206	164167	181181	138140	148160	158158	171171	180192	168174
blue	7	C8V20	197200	135164	166181	132140	148176	158158	171186	180192	170182
blue	7	C8V21	206206	152158	166181	132142	150162	158158	171171	180180	170174
blue	7	C8V22	197197	129138	166181	140150	150158	158168	168171	180180	180182
blue	7	C8V23	197197	129129	181181	132138	170178	158158	171171	180192	170172
blue	7	C8V24	200200	129164	166178	132136	160160	168168	168171	180192	184184
blue	7	C8V25	197197	129132	166175	138138	168178	158158	168171	180192	170174
blue	7	C8V26	200212	161164	166166	132132	148150	168168	171171	180192	170180
blue	7	C8V27	194197	120120	181181	132132	160160	168168	171171	180180	180180
blue	7	C8V28	197200	164176	166175	132140	148150	158168	168171	180183	168176
blue	7	C8V29	197200	161164	178186	140140	160160	168168	171171	192192	176182

red	15	A23R12	1	197197	129141	166175	156156	152160	188188	171171	180192	178178
red	15	A23R13	1	197197	135135	166181	132136	160160	166166	171171	180192	168188
red	15	A23R14	1	197200	155155	166181	132138	150150	166166	171171	180180	168168
red	15	A23R16	1	197200	120129	181181	138140	148156	158158	168171	192192	170184
red	15	A23R17	1	197197	129170	0	138138	148160	158158	168186	180180	170172
red	15	A23R18	1	197197	126150	175181	132138	160160	0	171171	180180	172174
red	15	A23R19	1	0	144173	0	132132	148160	160168	168186	180180	176184
red	15	A23R20	1	197197	150158	181181	132138	158156	170170	171171	180180	176178
red	15	A23R21	1	197200	155161	166181	138140	160160	0	171177	183180	0
red	15	A23R22	1	197200	129161	175175	138144	162162	172172	171171	180180	171184
red	15	A23R23	1	197200	132173	166166	132138	160160	158158	171171	195195	172184
red	15	A23R24	1	197200	161173	181183	132132	160164	158158	171171	180192	170184
red	15	A23R25	1	197197	132144	166175	132138	148174	0	171174	180192	168190
red	15	A23R26	1	197197	161164	181181	132138	148160	174174	171186	192192	178184
red	15	A23R27	1	197200	132150	175181	132158	172172	0	171186	180180	192192
red	15	A23R28	1	206206	155170	181181	138138	148148	170170	168171	180180	160172
red	15	A23R29	1	0	117129	178181	140140	164164	158180	171171	180180	170178
red	15	A23R30	1	197197	170179	0	132140	148148	172172	168171	180192	174174
red	15	A23R40	1	197197	129158	166181	132140	178178	168168	168171	192195	168170
red	15	A23R41	1	197206	150150	166181	136138	148150	158158	171186	180195	180180
red	15	A23R42	1	197200	170173	163163	132136	160174	158158	171171	180192	170192
red	15	A23R43	1	200206	150161	166166	136138	150174	158188	0	0	172180
red	15	A23R44	1	197197	152164	166181	132136	150152	158158	171171	180192	170180
red	15	A23R45	1	197197	129150	181183	138160	150152	166166	168186	180183	172174
red	15	A23R46	1	0	129129	0	140142	160160	160160	168171	180180	178178
red	15	A23R48	1	197200	170173	175181	132132	150164	0	171186	180180	172176
red	15	A23R49	1	197200	152176	166181	0	0	0	168174	180192	172172
blue	16	A23V15	2	197200	173173	175181	138156	150176	158158	171171	180180	174174
blue	16	A23V16	2	197197	129158	166181	136138	150178	158158	171171	180180	174186
blue	16	A23V17	2	197200	135141	166175	132140	148150	158158	171177	180180	172174
blue	16	A23V18	2	197206	173173	175181	132138	152160	158158	168171	180192	170170
blue	16	A23V19	2	197197	161161	166181	140140	160162	168172	171174	180192	184184
blue	16	A23V20	2	197197	132141	166175	138140	148148	158158	171186	192192	172172
blue	16	A23V21	2	197197	155158	172181	138138	156160	166166	171186	180195	170174
blue	16	A23V22	2	197197	141158	175175	132140	162178	176176	0	180192	172192
blue	16	A23V23	2	197200	141152	175178	140140	160164	158158	171186	192192	170184
blue	16	A23V24	2	197206	158167	178181	140160	178178	188188	0	180180	172184
blue	16	A23V25	2	0	132152	181181	138142	160160	158158	174186	180180	182184
blue	16	A23V26	2	197197	132132	166175	0	158164	158158	168171	180180	168188
blue	16	A23V27	2	197197	0	175181	136140	148150	166166	171171	192192	174180
blue	16	A23V28	2	197200	129141	175181	132156	148148	158178	168171	192192	184184
blue	16	A23V29	2	197200	129150	181183	138160	154178	158158	171171	180180	176190
blue	16	A23V30	2	197197	141147	175181	132138	148178	158158	0	0	0
blue	16	A23V40	2	200206	132164	175181	140156	148160	158158	168171	195195	170172
blue	16	A23V42	2	191200	158173	175181	138138	148160	158168	171171	180180	176186
blue	16	A23V43	2	203206	161182	166166	138158	178178	0	174186	180180	186186
blue	16	A23V44	2	200200	152155	181181	132138	178182	166166	168171	180183	0
blue	16	A23V45	2	0	164164	166166	138140	162178	158158	171186	192192	170178
blue	16	A23V46	2	0	132132	166175	136138	170172	158158	171171	180192	0
blue	16	A23V47	2	197197	164170	169181	132142	160160	168172	168171	180195	168172
blue	16	A23V48	2	197200	132135	166181	138152	148150	172172	171186	180180	172172
blue	17	B23R31	3	188200	138150	175181	132138	168178	158158	171171	180192	180180
blue	17	B23R32	3	197197	135138	166181	132132	158178	172172	171171	180180	182182
blue	17	B23R33	3	191197	164170	181181	140156	150162	158168	0	180192	182184
blue	17	B23R34	3	200206	129132	166181	132158	160160	158158	171177	180192	172186
blue	17	B23R35	3	191206	144161	178181	136140	150158	0	168168	180180	188188
blue	17	B23R36	3	197206	135173	175181	132138	162162	158158	168171	180192	168174
blue	17	B23R37	3	200200	152170	175181	132132	160172	174174	171186	180183	172182
blue	17	B23R38	3	188197	129129	166181	132138	158160	0	171186	180180	170170
blue	17	B23R39	3	197206	129129	175178	138138	148160	158182	171171	180180	174174
blue	17	B23R40	3	194200	129150	178181	138158	174174	160170	168168	183183	170174
blue	17	B23R41	3	197197	135135	166181	140142	150164	158158	171171	192192	170170
blue	17	B23R42	3	197206	129147	175175	138138	172178	158158	171171	180192	170172
blue	17	B23R43	3	197197	129129	178181	132138	148148	166166	171171	180180	170174
blue	17	B23R44	3	197200	117167	166175	134138	148148	158158	171186	180192	168172
blue	17	B23R45	3	197206	135147	181181	130136	156160	166166	168168	180192	172174
blue	17	B23R46	3	197197	132150	166175	136138	148162	158158	171171	180192	170176
blue	17	B23R47	3	197197	117173	175181	140142	162168	166166	171171	180180	170172
blue	17	B23R48	3	197197	150167	166175	136138	160162	174174	171186	180180	0
blue	17	B23R49	3	197200	129173	166175	138140	148160	158158	171171	180180	170170
blue	17	B23R50	3	197197	129164	175178	136138	152160	158158	171171	180183	172174
blue	17	B23R51	3	197197	132135	166181	138138	148160	176176	171186	183183	162174
blue	17	B23R52	3	197197	141141	166181	132140	162176	176190	171186	180180	168174
blue	17	B23R53	3	200200	138173	169175	132132	160160	168168	171186	180192	176188
blue	17	B23R54	3	197200	138164	166175	0	160160	158158	171186	192192	174178
blue	17	B23R55	3	197197	132132	166166	132140	162162	158158	0	180192	170180
blue	17	B23R56	3	194200	120132	169169	134140	164174	158166	171186	180195	174174
blue	17	B23R57	3	197197	132150	175181	128138	164178	148158	174186	180180	172172
blue	17	B23R58	3	197200	129150	166166	138156	150160	0	171171	180180	176180
blue	17	B23R59	3	197197	132161	166175	132142	162162	168168	168171	180180	178178
blue	17	B23R60	3	200206	135138	166169	132132	156178	158158	171171	180192	176178
red	3	B23V01	4	197206	129129	178181	132138	148156	166166	171171	180192	170172
red	3	B23V02	4	197197	170176	181181	132140	162162	160168	171186	180192	172182
red	3	B23V03	4	197197	129164	181181	132140	178178	188188	171171	180180	174174
red	3	B23V04	4	197197	144155	166181	132156	164164	160170	171186	180180	174180
red	3	B23V05	4	197197	129138	181181	132138	148150	166166	168171	180180	180194
red	3	B23V06	4	197197	129138	166181	136136	150152	158158	171171	180192	172182
red	3	B23V07	4	197206	152161	175181	132140	156156	174174	168189	180192	170188
red	3	B23V08	4	200206	161161	166183	132138	160172	176190	171171	180180	170170
red	3	B23V09	4	197197	126161	181181	132138	156160	158176	171171	180195	170172
red	3	B23V10	4	197197	167170	175181	138138	150162	158174	171186	180180	172174
red	3	B23V11	4	197197	135135	166178	140156	152174	166166	168174	180192	172174
red	3	B23V12	4	197197	129132	166181	138140	150150	158158	171177	183195	174180
red	3	B23V13	4	197200	132150	166175	138140	162178	158158	171171	180192	170176
red	3	B23V14	4	200200	132170	175181	132132	158158	158176	168171	180192	168178
red	3	B23V15	4	197197	129170	166178	132138	178178	0	171171	180192	170170
red	3	B23V16	4	197206	173173	181181	132138	158162	178182	0	180192	170178
red	3	B23V17	4	197200	129129	175175	138138	148150	182182	168171	180180	172176
red	3	B23V18	4	197200	150164	166178	132156	162162	174176	168171	180192	166180
red	3	B23V19	4	197197	129132	166181	156162	148148	188188	171186	180180	168170

red	3	B23V20	4	197200	126129	166175	132138	160164	166172	171171	180180	170178
red	3	B23V21	4	206206	129129	166166	132138	172172	166166	171171	180192	168178
red	3	B23V22	4	197197	135144	181181	138140	148160	158158	171186	180180	174184
red	3	B23V23	4	197197	173176	175181	0	176176	174174	168168	180192	170188
red	3	B23V24	4	197212	132132	166175	132138	174174	166166	168186	192192	172188
red	3	B23V25	4	197200	129144	175181	138138	160160	168168	168171	180180	170170
red	3	B23V26	4	197206	161176	166169	132138	164166	168168	171171	180180	178178
red	3	B23V27	4	197200	170170	166181	132138	166166	158158	171186	180180	170184
red	3	B23V28	4	194197	144167	178181	138138	148160	158176	168186	192192	172172
red	3	B23V29	4	197197	129161	175181	128160	160160	168168	171171	180180	170174
red	3	B23V30	4	197200	150167	166181	138160	170170	158158	186186	195195	170186
red	4	C23R01	5	197200	164173	166166	140140	150150	182182	171171	180192	170174
red	4	C23R02	5	197197	132152	175181	136160	152156	168168	171186	180180	172188
red	4	C23R03	5	197197	129161	175181	132138	164176	156166	171186	192192	172172
red	4	C23R04	5	197200	132161	181181	132140	160160	174188	171186	0	174174
red	4	C23R05	5	197197	123152	166166	132140	150158	160168	171171	180192	178178
red	4	C23R06	5	197200	129158	181181	130130	156160	158158	177186	180180	172184
red	4	C23R07	5	206206	129129	166181	132140	148160	178178	177177	180183	178184
red	4	C23R08	5	197197	164167	166181	138138	150160	148158	177177	192192	186186
red	4	C23R09	5	197197	123141	175175	130132	152162	172172	171171	180183	170174
red	4	C23R10	5	200200	129144	166166	132140	160162	156166	171186	180180	170170
red	4	C23R11	5	197197	173173	0	128138	150150	164164	171171	183192	184188
red	4	C23R12	5	191191	135158	166166	138146	162176	182188	171174	192192	170184
red	4	C23R13	5	200200	117117	166166	132132	148160	188188	171171	192192	176176
red	4	C23R14	5	197197	129138	0	132132	148150	158158	171171	180180	172190
red	4	C23R15	5	194200	150164	181181	132132	150150	188188	168171	192192	180180
red	4	C23R16	5	197197	0	0	132140	162162	188188	186186	180192	168168
red	4	C23R17	5	197197	129129	166175	132132	148148	184188	171171	192192	168168
red	4	C23R18	5	197200	135155	175175	140140	148176	158158	171186	180180	182182
red	4	C23R19	5	197197	132161	175183	132132	160176	168168	171186	0	170170
red	4	C23R20	5	191197	129129	166166	132136	148148	148148	171171	180192	172172
red	4	C23R21	5	197197	129129	175175	136140	164176	158168	0	180192	172182
red	4	C23R22	5	197200	129132	175175	132136	160176	168168	168171	180180	186192
red	4	C23R23	5	197197	167173	175181	136136	150162	168168	171186	180183	0
red	4	C23R24	5	197206	167176	166181	0	176176	160166	168171	180192	0
red	4	C23R25	5	197197	129144	166181	134158	156160	160166	171171	192195	172172
red	4	C23R26	5	197200	0	178178	134138	148176	158172	186186	180192	170176
red	4	C23R27	5	197197	135167	166181	132132	152180	158158	171171	180192	168168
red	4	C23R28	5	197212	138170	166181	132132	150160	158182	171171	180180	170170
red	4	C23R29	5	206212	129147	181181	0	158158	158158	0	180192	0
red	4	C23R30	5	191206	120129	166175	156156	162162	158158	168168	180183	170170
red	4	C23R31	5	197197	129167	166166	138138	160178	156166	171171	180180	182182
blue	7	C23V01	6	197197	135173	178181	132132	0	0	171186	180180	168178
blue	7	C23V02	6	197197	129132	166175	0	164164	148158	171171	180192	170178
blue	7	C23V03	6	194197	158161	166181	138138	152178	158158	168186	192192	170170
blue	7	C23V04	6	197206	132164	166166	140140	160160	158158	171171	180180	170174
blue	7	C23V05	6	197197	152176	181183	132138	150156	158176	186186	0	170182
blue	7	C23V06	6	200200	161167	181181	132140	150176	170170	186186	192192	170172
blue	7	C23V07	6	197200	132167	166175	140140	156160	158178	177177	0	170170
blue	7	C23V08	6	200200	129164	166175	132140	0	168168	171177	180180	170170
blue	7	C23V09	6	197200	167170	181181	132132	150150	168168	168171	180192	170178
blue	7	C23V10	6	200200	144150	166181	132132	148160	152166	171171	180192	170188
blue	7	C23V11	6	200200	129132	0	132154	178178	166166	171186	180192	176176
blue	7	C23V12	6	197200	117132	181181	132132	148148	160160	171186	180192	170178
blue	7	C23V14	6	197200	147164	166178	136136	156172	172176	171186	195195	178178
blue	7	C23V16	6	197206	132147	166181	132140	148148	146158	171186	192192	170180
blue	7	C23V18	6	197200	132147	166175	138138	160176	148158	171171	180192	170176
blue	7	C23V20	6	197197	158158	0	138138	150158	182182	186186	180183	170172
blue	7	C23V22	6	197197	170170	166166	0	148150	166166	168171	192192	170170
blue	7	C23V24	6	197197	129129	166181	134134	148176	174174	171171	180180	170170
blue	7	C23V26	6	197197	176176	166175	132132	162178	148152	171186	180183	178178
blue	7	C23V28	6	197197	129170	0	138146	162176	158158	171171	180195	168168
blue	7	C23V30	6	197206	144155	166175	132138	148176	158158	171186	180183	172172
blue	7	C23V31	6	197200	164164	175181	140140	156172	176176	171171	183195	172178
blue	7	C23V32	6	197197	135158	166181	132132	150150	160160	171171	192195	170170

red	15	A25R14	0	132161	166175	132140	150174	162162	171171	180180	176178
red	15	A25R15	197200	150155	175181	138156	160160	158182	168171	180192	172182
red	15	A25R40	0	129141	181181	138142	161176	0	171171	192192	176188
red	15	A25R41	197200	161173	175181	132140	150160	158158	171171	180192	172172
red	15	A25R52	0	132138	0	132138	178178	158158	171186	180180	170174
red	15	A25R42	197200	152161	166175	132140	178178	176176	168171	180192	170170
red	15	A25R43	197200	147155	166175	132138	162162	158166	171186	180195	172172
red	15	A25R44	0	152179	166181	138142	178178	158158	168171	192195	172172
red	15	A25R45	197206	158164	175181	132142	164176	166166	168171	180180	168172
red	15	A25R46	200200	173173	181181	132142	160160	0	171171	180192	172182
red	15	A25R47	197200	129150	175175	132138	148160	158158	171186	192192	168168
red	15	A25R48	197200	129129	175181	132138	160160	158176	171186	0	170174
red	15	A25R49	0	129173	166181	140140	148178	160160	171171	180195	172178
red	15	A25R50	206206	150150	166166	138138	148148	188188	171171	180180	170172
red	15	A25R51	197200	129129	181181	136138	160160	0	171171	192192	170178
red	15	A25R53	197197	155155	181181	140140	160160	176176	171171	180180	176176
red	15	A25R54	197200	117173	166181	132138	148150	182182	169171	180180	174188
red	15	A25R55	200200	144144	175181	132138	148160	158158	171171	180192	168168
red	15	A25R56	0	129132	166181	132132	162162	162162	186186	180183	0
red	15	A25R57	197197	144144	166181	138138	160180	170170	171186	180180	170174
red	15	A25R58	206206	132144	175181	140156	162162	158158	168186	183192	170176
red	15	A25R59	197200	152161	172181	132132	150160	164164	168171	180180	170188
red	15	A25R60	197197	120129	181181	138140	178178	162162	171171	180183	184184
red	15	A25R61	197200	164164	175175	0	0	0	171171	180180	170174
red	15	A25R62	0	129170	166181	0	150160	0	171171	192192	168180
red	15	A25R63	197197	132176	166183	132138	148160	160160	168186	180192	170176
red	15	A25R64	197197	161170	181181	0	0	0	168171	180192	172180
red	15	A25R65	188197	155164	181181	132138	148162	158178	186186	195195	168174
red	15	A25R66	197200	138161	175181	132138	176176	160160	171186	192195	178176
red	15	A25R67	197200	129132	166166	138138	148178	160160	168186	180180	0
red	15	A25R68	200212	147164	175181	132138	150158	158166	171177	180195	170178
red	15	A25R69	197197	129132	178181	138138	150160	0	171171	180195	168170
blue	16	A25V40	200200	126150	175175	132140	150150	168168	171171	180180	174188
blue	16	A25V41	197200	129155	175181	132140	160162	158158	171186	180180	176178
blue	16	A25V42	197197	132164	166175	138138	164164	164164	171177	180180	170186
blue	16	A25V43	206206	152158	166166	132146	164176	172176	171186	180192	174174
blue	16	A25V44	197197	132173	166181	138140	158176	182182	171186	180180	176176
blue	16	A25V45	197197	132138	175181	136138	178178	158158	171171	180180	168172
blue	16	A25V46	197197	158173	166181	140144	160160	0	168171	180192	176186
blue	16	A25V48	0	173179	172181	136136	162162	0	171177	180195	168170
blue	16	A25V49	200203	152158	166181	132138	160174	168168	168171	180192	172174
blue	16	A25V50	0	129158	169181	138138	150162	0	171177	180180	178182
blue	16	A25V51	197200	132150	175183	132138	148150	0	168171	180180	198198
blue	16	A25V52	197197	126161	181181	132138	160178	172172	171171	192192	182188
blue	16	A25V53	197200	147170	181183	132140	158160	166166	171171	180180	168174
blue	16	A25V55	197200	132135	166178	138138	160164	176176	171174	180180	168180
blue	16	A25V56	197206	152161	166166	138142	150176	158158	168186	180192	172172
blue	16	A25V57	0	161161	166181	138142	154154	164176	171186	192192	0
blue	16	A25V58	197197	129164	0	132138	150150	158158	171171	180180	170186
blue	16	A25V59	197200	152161	166175	140140	156162	176176	171171	180195	178178
blue	16	A25VD9	197200	117138	178181	138142	152152	158158	168168	180192	172172
blue	16	A25VH3	197206	132179	175181	132138	150178	158158	171177	180180	172174
blue	16	A25V61	197206	129129	166175	138140	150150	158158	168171	180180	174174
blue	16	A25V63	188206	152155	166166	136138	150178	166166	177186	192192	170170
blue	16	A25V65	197197	129173	166181	140140	174178	170170	168186	192192	170172
blue	16	A25V66	197197	150173	178178	132138	150150	158158	171171	0	172172
blue	16	A25V67	197206	132158	166181	132132	156160	178178	168171	180192	170170
blue	16	A25V68	197197	138167	175181	132132	150160	170170	168186	180180	180182
blue	16	A25V69	197212	150152	166183	132138	148178	166166	171177	180195	174178
blue	17	B25R31	197197	132132	166166	136138	148160	162172	171171	180192	170170
blue	17	B25R32	197206	129129	166166	138142	150158	154164	168171	180195	170170
blue	17	B25R33	197206	152170	175181	134140	178178	158174	171186	180192	170184
blue	17	B25R34	191206	132132	166181	132138	148148	172172	171186	180195	174182
blue	17	B25R35	197200	129138	166178	132142	150160	158158	171171	180192	162174
blue	17	B25R36	200200	147147	166175	132132	178178	160176	171177	192195	168168
blue	17	B25R37	197197	129167	166166	132138	160172	170170	171186	183192	170174
blue	17	B25R38	197200	141158	166166	140156	150178	166166	0	180192	184184
blue	17	B25R39	197197	129167	166166	142148	148150	158172	171171	180180	174180
blue	17	B25R40	197200	138138	181181	132148	148148	158188	168168	180180	170170
blue	17	B25R41	200200	152158	166166	140140	152152	162162	171171	180180	170182
blue	17	B25R42	197197	155155	181181	132132	160178	166166	171186	180180	184184
blue	17	B25R43	200206	129129	175178	132132	160178	158158	171177	192192	170170
blue	17	B25R44	197200	129173	166181	132132	158178	0	171186	180192	170170
blue	17	B25R45	197197	138164	181183	136138	150178	176190	168171	180192	174178
blue	17	B25R46	197197	147152	166166	132136	174176	158166	168171	192192	174180
blue	17	B25R47	197197	129167	166166	140142	148150	168168	168168	180180	170176
blue	17	B25R48	200200	173173	175181	132156	152152	172172	171171	180195	176176
blue	17	B25R49	197200	144144	166166	138160	160160	158158	171186	180180	170172
blue	17	B25R50	197197	144173	166181	140140	150160	158188	171171	180192	174178
blue	17	B25R51	197197	158164	175175	132140	156178	168172	171171	180180	170182
blue	17	B25R52	197206	132132	175181	132140	158178	158172	168171	180192	168178
blue	17	B25R53	197197	158161	181181	138138	148160	0	168171	192192	170176
blue	17	B25R54	197197	132161	175181	140158	148148	158158	171171	180192	174192
blue	17	B25R55	197197	135164	166166	138156	150160	158158	168171	180192	170178
blue	17	B25R56	197197	135150	166172	132138	160170	0	171171	180192	168174
blue	17	B25R57	197197	150150	166181	140140	160160	158158	171171	192192	170184
blue	17	B25R58	200200	132132	166166	132138	158178	178178	171177	180195	168172
blue	17	B25R59	197197	129132	166166	138140	150178	158176	171171	180180	172174
blue	17	B25R60	197206	141164	166181	130138	156156	164174	168186	180180	174176
red	3	B25V01	197206	129147	175181	140146	178178	158158	177186	192192	170170
red	3	B25V02	197206	132132	166183	132138	150158	158158	168171	180192	174182
red	3	B25V03	197197	150150	166181	140150	152162	158158	0	180195	180186

red	3	B25V04	197197	132150	166181	132138	164164	158158	168171	192192	178180
red	3	B25V05	197197	129161	166181	138148	150162	158164	174186	180192	176188
red	3	B25V06	197200	161170	181181	132162	164176	176176	174186	180180	172178
red	3	B25V07	197197	155158	166181	140140	150162	158158	171171	180180	0
red	3	B25V08	197200	152164	175181	132140	148150	158158	177177	183183	170176
red	3	B25V09	188197	167167	181181	132132	148178	158158	171171	180180	168174
red	3	B25V10	197197	135150	181181	136138	178178	168168	171171	180180	170176
red	3	B25V11	197200	135150	175181	132140	162178	158158	171171	183192	172172
red	3	B25V12	197206	132176	175181	140150	148164	158158	171177	180180	172176
red	3	B25V13	197197	132147	175181	132138	166166	158158	171171	183183	176178
red	3	B25V14	197200	135150	166183	132132	150158	168168	168171	180183	170172
red	3	B25V15	197200	129141	181181	134140	150150	158168	171186	180192	174174
red	3	B25V16	197200	129150	178181	138142	148160	158158	171171	180180	170174
red	3	B25V17	197200	152152	181181	134140	150150	166172	168186	180180	188188
red	3	B25V18	197200	144152	166181	138158	150160	176188	168171	180192	172180
red	3	B25V19	197197	132132	166166	134140	156156	158158	171186	180189	162174
red	3	B25V20	197200	135176	166181	132138	152162	166166	171171	183183	172176
red	3	B25V21	197200	158170	175181	140144	150178	158158	174174	180180	174176
red	3	B25V22	197200	129129	166166	132140	172174	158166	171171	180192	172176
red	3	B25V23	197197	129129	166166	140144	150158	158158	171171	180180	170186
red	3	B25V24	206206	135135	172181	132138	162178	158168	171186	180180	182190
red	3	B25V25	197200	129132	175181	132136	160164	158158	171174	180180	172192
red	3	B25V26	197212	132132	166181	132146	150170	158158	183186	180195	170170
red	3	B25V27	197197	132132	166178	138142	156158	158158	168171	180180	174176
red	3	B25V28	197200	129152	166175	132132	162178	158170	171171	180195	170170
red	3	B25V29	197206	129129	175175	138138	160164	166166	171186	180180	170172
red	3	B25V30	197197	132132	166181	140140	150156	158176	168171	192195	170184
red	4	C25R01	197200	158158	166181	132136	150150	148158	171171	180192	0
red	4	C25R02	191197	132135	166181	132134	150160	176176	171186	192192	170174
red	4	C25R03	197200	132144	166181	138138	150150	172172	171186	180192	0
red	4	C25R04	197197	152161	166166	132132	150158	170170	171186	180192	168168
red	4	C25R05	200200	135152	166175	138138	150160	148148	171177	180180	170174
red	4	C25R06	197197	144164	181181	142142	146146	158168	171177	180192	170174
red	4	C25R07	197197	147161	181181	138138	146146	158158	168171	192195	174186
red	4	C25R08	0	155155	166181	132140	156156	148160	168171	192192	170174
red	4	C25R09	197200	155155	166175	132140	150150	178178	171186	180192	170172
red	4	C25R10	197203	132161	166166	140144	150150	158158	168171	180192	168174
red	4	C25R11	0	132152	166181	132134	148150	158158	171177	180183	174174
red	4	C25R12	197197	132132	166181	132132	148178	158164	171171	180192	168178
red	4	C25R13	200200	164167	175181	140140	150160	0	168177	192192	170170
red	4	C25R14	197200	132138	166166	132134	148148	168168	171171	192192	174182
red	4	C25R15	0	129161	175175	132132	148162	172172	171186	180192	168180
red	4	C25R16	197197	123147	166175	140140	156160	164164	171171	180192	168170
red	4	C25R17	206206	129150	166175	132138	156170	164164	168171	192192	168172
red	4	C25R18	0	173173	166183	132140	162162	158158	168186	180180	168174
red	4	C25R19	200200	161173	181186	132138	148160	158164	171171	180192	174190
red	4	C25R20	0	138150	175181	138138	176176	158164	174186	180180	180186
red	4	C25R21	200206	129164	169181	132138	150184	158158	0	180180	170180
red	4	C25R22	200206	152167	169181	138138	160174	166166	171177	180192	170174
red	4	C25R23	200200	129132	178181	138138	150150	158164	171186	180192	170176
red	4	C25R24	197197	129155	166181	132138	150176	158158	171171	180195	170174
red	4	C25R25	197200	161161	166181	140140	150180	158164	171171	180180	172172
red	4	C25R26	0	132161	166181	132132	170170	158164	171186	180180	172174
red	4	C25R27	206206	0	0	132132	158160	172172	171171	180180	172172
red	4	C25R28	200200	132161	175175	132138	162178	168168	171171	180180	170178
red	4	C25R29	188197	132132	166166	140140	160160	158158	171177	180180	172174
red	4	C25R30	200206	170173	175175	156160	156160	158164	177186	180192	170170
red	4	C25R31	197206	0	172175	132132	148162	158164	168171	180192	172184
blue	7	C25R32	200200	152152	172172	132150	158176	158164	168171	192192	170176
blue	7	C25R33	0	129155	166166	140142	162162	158164	171186	183183	168170
blue	7	C25R34	200206	161161	166175	132156	148162	168166	168171	192192	168176
